# A cytometric framework to assess trends in the morphological structure of bacterioplankton communities along freshwater environmental gradients

**DOI:** 10.1093/ismeco/ycaf223

**Published:** 2025-11-25

**Authors:** Sara Soria-Píriz, Paul A del Giorgio

**Affiliations:** Département des Sciences Biologiques, Carbon Biogeochemistry of Boreal Aquatic Systems (CarBBAS) group, Groupe de Recherche Interuniversitaire en Limnologie (GRIL), Université du Québec à Montréal, SB-2855, 2080 St-Urbain, Montréal, Québec, H2X 3X8, Canada; Département des Sciences Biologiques, Carbon Biogeochemistry of Boreal Aquatic Systems (CarBBAS) group, Groupe de Recherche Interuniversitaire en Limnologie (GRIL), Université du Québec à Montréal, SB-2855, 2080 St-Urbain, Montréal, Québec, H2X 3X8, Canada

**Keywords:** bacterioplankton, flow cytometry, morphological structure, lakes, environmental gradients

## Abstract

Bacterioplankton communities are characterized by varying distributions of cell size, shape and internal complexity, and macromolecular composition, yet there have been few attempts to quantitatively describe this complex community structure and to assess how it varies among communities and habitats. Here we present a framework to assess this morphological structure, based on the analysis of dot clouds resulting from flow cytometric measurements of side and forward scatter and cell fluorescence of individual bacterioplankton cells. Each community has a characteristic cytometric dot cloud, which forms an ellipsoid that can be described by a combination of metrics that quantify its shape, elongation, volume, orientation, and internal complexity. We apply this framework to assess how the bacterioplankton morphological structure (BMS) varies in 637 lakes distributed across Canada, covering a wide range of limnological, watershed, and climatic features. We show that there is a BMS core, which is characterized by small, simple and oblate shapes, and low overall fluorescence i.e. present in all lakes but is prevalent in oligotrophic lakes with hydrologically less evaporated water and low retention time, likely reflecting mass effects and allochthonous bacterial inputs. We further show that along gradients of increasing network water residence time, system productivity and dissolved organic carbon enrichment, there is a clear succession wherein BMS becomes increasingly dispersed, complex, and prolate shapes, likely reflecting environmental selection of aquatic taxa.

## Graphical Abstract

**Graphical Abstract:**
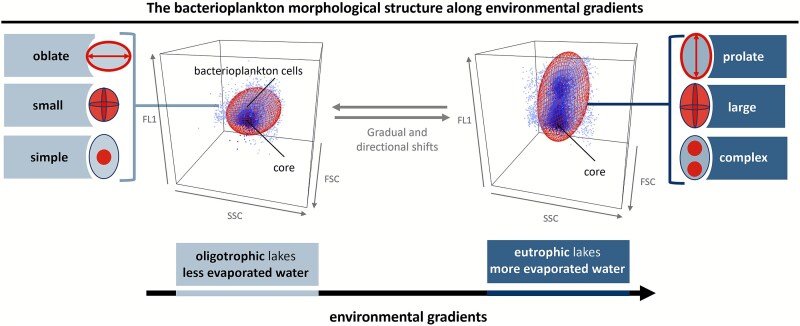

## Introduction

Flow cytometry has been one of the most used high throughput, high resolution techniques to analyze microbial communities over the past decades [1–5]. A cytometric analysis typically results in a cloud of points in by-plots of these various cytometric parameters, and these clouds reflect the collective cytometric properties of the individual cells that make up the community. In medical and cell biology applications, there has been a long-standing recognition that the “shape” of these cytometric dot clouds, and not just the individual cytometric parameters, is of importance to discriminate between various populations of cells, different physiological states of the same cells or even to diagnose some diseases [6, 7]. In contrast, cytometric protocols used to assess microbial communities inhabiting the various aquatic and terrestrial ecosystems have generally focused less on the shape of the clouds and more on the magnitude of the individual cytometric parameters, that includes 90° light side scatter of cells (SSC, a proxy of the complexity and granularity of the cell), forward side scatter (FSC, a proxy of the size cell), red or orange autofluorescence associated to photosynthetic pigments of autotrophs, and the fluorescence conferred by a wide range of fluorochromes, e.g. green fluorescence from DNA specific fluorochromes used to stain bacterioplankton (FL1, a proxy of nucleic acid content of the cell) [8]. For example, studies assessing autotrophic pico- and nanoplankton in lakes and oceans separate various potential populations based on their distinct scatter and fluorescence signatures and typically seek to characterize these populations on the basis of their average fluorescence and determine diversity index [9–13].

Most studies assessing heterotrophic bacterioplankton typically quantify the total number of stained bacterial cells that fall above a certain threshold of detection of either side scatter or fluorescence. Some studies have further identified different bacterial fractions within the cloud of cells, and in aquatic ecosystems, two recurrent fractions have emerged: cells with high nucleic acid content (HNA cells) and cells with low nucleic acid content (LNA cells), based on the magnitude of DNA-associated fluorescence [14–16]. Other studies have gone a step further, and have applied automatic classification methods and machine learning to classify bacterial subgroups within samples [17], to derive indices of community diversity [13, 18–20], and to combine flow cytometry data with 16S rRNA gene amplicon sequence data to associate bacterial taxa to functional groups [21].

What few studies to date have done is to assess how the actual shape of the cytometric dot clouds, which define the morphological structure of the communities analyzed, varies among microbial communities in different habitats and along environmental gradients. This morphological structure integrates various aspects of the bacterioplankton community, because it accounts for size and shape, internal cell complexity, and the internal nucleic acid (DNA and ARN) contents, which itself is linked to multiple other facets of cell physiological state, growth, and function. The morphological structure therefore may provide insights that cannot be obtained by any other single approach: physiological essays such as bacterial production or nitrogen fixation measurements provide information on community performance and function; genomics provide information on taxonomic and phylogenetic composition and functional capacities, and microscopy may provide information on cell abundance and size, but none of these approaches provide the type of high throughput, integrative information needed to map these community distributions of cell sizes and related morphologic or phenotypic features.

The morphological structure derived from cytometric dot clouds is therefore a critical dimension of microbial communities that lies at the interface between community composition and taxonomic structure, community functional capacities, physiological traits and responses, and overall metabolic performance. It represents the physical and macromolecular fingerprint of these communities, which captures variation in key cellular features that reflect how bacterioplankton communities structure as a function of major environmental and ecological drivers. This fingerprint, however, is largely missing from most microbial studies, mostly because there are no consistent approaches to quantify this dimension of microbial communities. In this paper, we develop a framework to quantify the bacterioplankton morphological structure (BMS hereafter), defined by a cytometric structure which is based on a series of metrics derived from SSC, FL1 and FSC parameters. We applied this framework to characterise the BMS in 637 lakes that are part of the pan-Canadian LakePulse project, spanning a continental scale, from the Arctic to the Atlantic Ocean basins and broad environmental and climatic gradients, and we further linked the various aspects of the BMS to geography, environment, hydrology and lake features.

## Material and methods

### Area description and sampling collection

A total of 664 lakes were sampled once across Canada over three summers (2017, 2018, and 2019) from the beginning of July to the end of August, as part of the Natural Sciences and Engineering Research Council of Canada (NSERC) Canadian Lake Pulse Network [22]. The study area extended over the five continental basins, i.e.: Atlantic Ocean, Great lakes-St. Lawrence, Hudson Bay, Arctic Ocean and Pacific Ocean, and covering a wide range of geographical distance, watershed landscape, lake morphometry, hydrology and environmental characteristics [22]. The complete field protocol information is provided by [23].

### Environmental dataset

Environmental data collection was classified in four categories.

*Geographical and climate* category includes air temperature, wind average and relative humidity were measured on site; precipitation recorded over the previous 30 days before the sampling collection (precipitation) was obtained from ERA5-Land hourly data from 1950 to present [24], and lake altitude derived from HydroLAKES v. 1.0. [25]. *Watershed* category includes watershed land use fractions and natural land cover fractions, derived from a 30 m resolution remote sensing dataset as described in [22]. *Lake morphometry* category included lake watershed area, watershed slope within 100 m of the shoreline, lake area, lake depth, lake volume, lake circularity, water discharge, water residence time and were also derived from HydroLAKES v. 1.0. [25]. Shoreline complexity, which defines the degree of variation in the shape and structure of the shoreline of a lake, and dynamic ratio, a metric that describes the lake shape, were determined according to [26]. *Limnological* category included chemical variables such as surface water temperature (Twater), pH, specific conductivity, salinity and dissolved oxygen (DO) which were performed on site (field protocols described in [23]. The ions concentrations of magnesium, potassium, calcium, sodium and chloride were determined following U.S. Environmental Protection Agency protocols (1994, 1997). Deuterium excess (d-excess), which represents a departure from the local meteoric signal and an index of the degree of evaporation in a given water sample was calculated following [27], dissolved organic carbon (DOC) concentration and dissolved inorganic carbon (DIC) concentration, chlorophyll *a* concentration, absorption of non-algal particles at 443 nm, total suspended solids, organic suspended particulate matter, mineral suspended particulate matter, total phosphorus (TP) total nitrogen (TN), the bacterial abundance (BA), and the spectral slope of the coloured dissolved organic matter (CDOM), which was calculated for the wavelength interval of 275 to 295 nm (S_275–295_) following [28], which represents a proxy of the CDOM molecular weight (MW), and source composition. Methodological details for the limnological variables are provided in Supplementary Information.

### Flow cytometric analysis

Unfiltered water was collected with an integrated tube sampler over the euphotic zone down to 2 m below the surface. Cryotubes (2 ml) were filled with 1.8 ml of unfiltered water, were preserved with 180 μL of 1% paraformaldehyde +0.05% glutaraldehyde solution (final concentration) and frozen at −80°C until flow cytometric analysis. Samples were analysed on a BD Accuri™ C6 flow cytometer (BD Biosciences, San Jose, CA, USA) equipped with a 20 mW 488 nm laser, a 14.7 mW 640 nm laser and a 70-μm nozzle. All samples were analysed at low flow rate (14 μL/min) and were recorded with a threshold set at 500 for FL1 to easily identify the background noise at the bottom part. Between 10 000–40 000 events were collected depending on the sample, and if they showed a rate > 2500 events s^−1^, samples were diluted with nanopure water to avoid coincidence. Data was collected at log scale. Fluorescent beads (Trucount™ Absolute Count Tubes, BD Biosciences, #340334) were run at the beginning, middle and end of each analysis day to assess instrument performance (flow rates and fluorescence scatter). The method described above is based on that published by [2]. Further details for the analysis are in Supplementary Information.

### Defining the bacterioplankton cytometric structure

**Figure 1:**
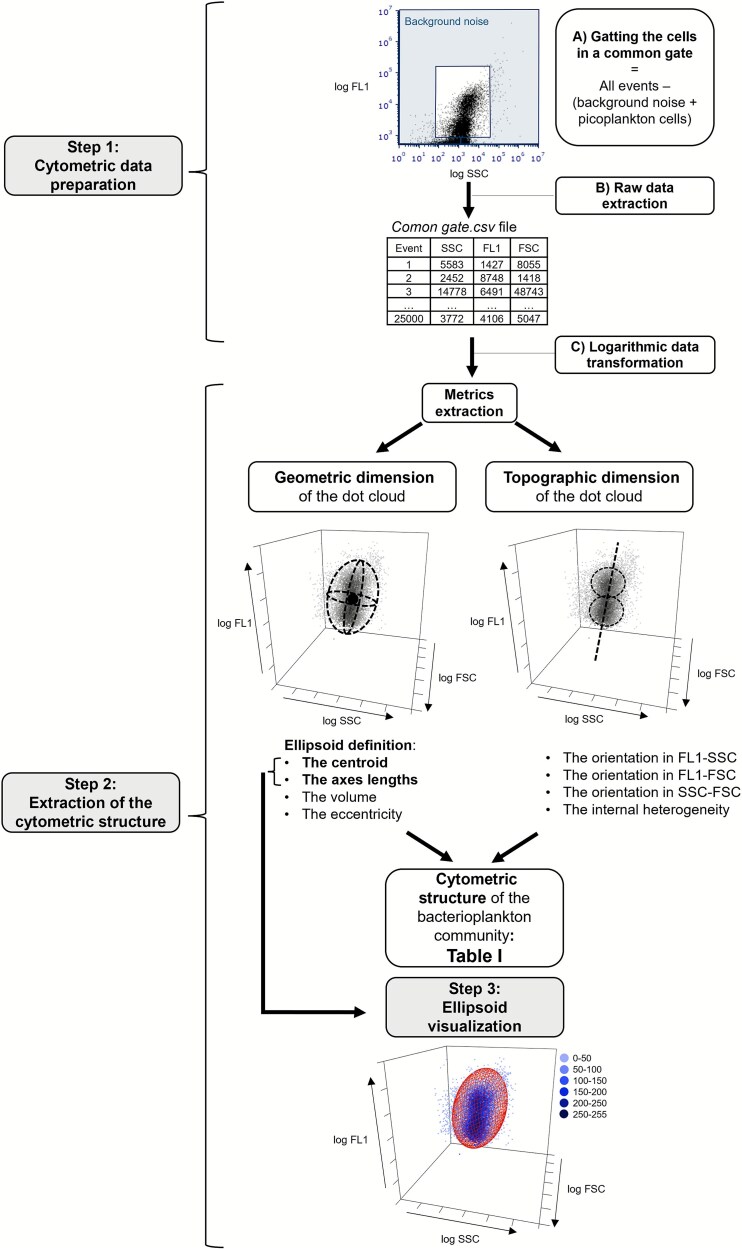
Defining the bacterioplankton morphological structure (BMS). Workflow showing the different steps to extract the BMS in freshwater samples.

Here we describe the steps involved in the definition of the cytometric structure to capture the BMS, following a workflow i.e. summarized in Fig. 1.

#### Step 1: cytometric data preparation

Bacterial cells of each sample were plotted in an SSC vs FL1 cytogram at logarithmic scale and gated using a single common gate for all samples, which was defined after checking all the samples by visual inspection to make sure that it encompassed the range of cytometric populations encountered (Fig. 1). The events retained within the common gate were those remaining after subtracting background noise, identified by inspecting green fluorescence vs side scatter cytograms, and cyanobacteria (photosynthetic picoplankton), the latter removed to avoid the overlapping with heterotrophic bacteria by inspecting the green fluorescence vs orange or red fluorescence cytograms (data not shown) (Step1A in Fig. 1). For each sample, raw data for SSC, FL1, and FSC parameters of the events within the common gate were exported as *.csv* file using FCS Express 6 (De Novo™ Software (Step1B in Fig. 1), and subsequently were logarithmic scale transformed (Step1C in Fig. 1).

#### Step 2: extraction of the bacterioplankton cytometric structure

A series of metrics were extracted to describe the geometric and topographic dimensions of the cytometric dot clouds, which altogether define the BMS (Table 1). For the geometric dimension, a 3-dimensional analysis was computed using the values of the SSC, FL1 and FSC considering a 90% covariance error ellipsoid (Fig. 1, Table 1). The centroid of the ellipsoid was defined by the means of SSC, FL1 and FSC values (centroid_SSC_, centroid_FL1_ and centroid_FSC_ respectively). The axes lengths of the ellipsoid were defined by the width (W) calculated in the SSC axis as $2\sqrt{6.251\times{\lambda}_{SSC}}$, the height (H), calculated as$2\sqrt{6.251\times{\lambda}_{FL1}}$ in the FL1 axis; and the length (L) calculated as $2\sqrt{6.2511\times{\lambda}_{FSC}}$ in the FSC axis, where ${\lambda}_{SSC}$, ${\lambda}_{FL1}$ and ${\lambda}_{FSC}$ correspond to the eigenvalues of the SSC, FL1 and FSC covariance matrix respectively and 6.251 correspond to the critical value from the chi-square distribution for a 90% confidence level with 3 degrees of freedom. The *ellipsoid volume* (V) was computed as $V=\frac{4}{3}\pi abc$, where *a*, *b* and *c* are the semi-axis, i.e. half of the W, H, and L, respectively. The *eccentricity*, which is a measurement of the ellipse elongation, and which varies from 0 (circular ellipse) to 1 (elongated ellipse), was determined in the FL1-SSC plane, using the W and H values and calculated as $e=\sqrt{1-\frac{b^2}{a^2}}$, where *e* is the eccentricity, *a is* the semi-major axis and *b* is the semi-minor axis of the ellipse.

**Table 1:** The cytometric metrics. Description of the cytometric dot cloud dimensions and their respective metrics using the values related to the 90° light side scatter (SSC), green fluorescence (FL1) and forward scatter (FSC) parameters.

**Cytometric cloud dimension**	**Metric**	**Abbrev.**	**Description**	**Conceptual diagram**
Geometric	Centroid	centroid_SSC_ centroid_FL1_ centroid_FSC_	Averages of SSC, FL1 and FSC	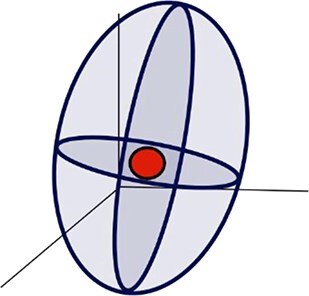
	Ellipsoid height	H	Dimension of the 90% covariance error ellipsoid in the FL1 axis	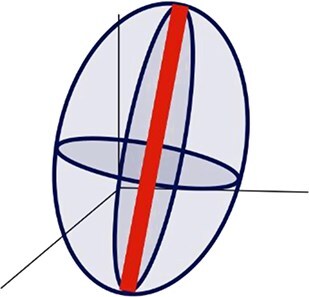
	Ellipsoid width	W	Dimension of the 90% covariance error ellipsoid in the SSC axis	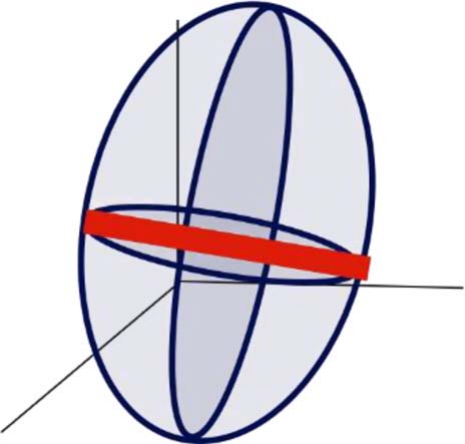
	Ellipsoid length	L	Dimension of the 90% covariance error ellipsoid in the FSC axis	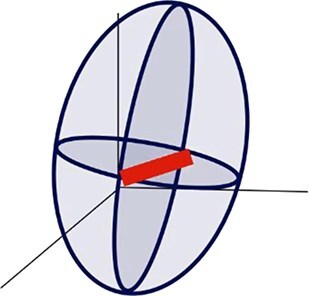
	Ellipsoid volume	V	Volume of an ellipsoid using the dimensions of the 90% covariance error ellipsoid.	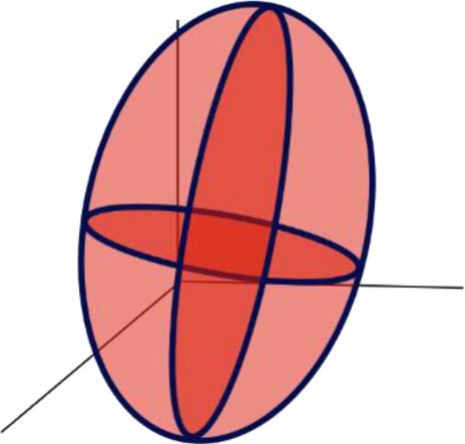
	Eccentricity	Eccentricity	A measure of the elongation of an ellipse using the SSC-FL1 plane	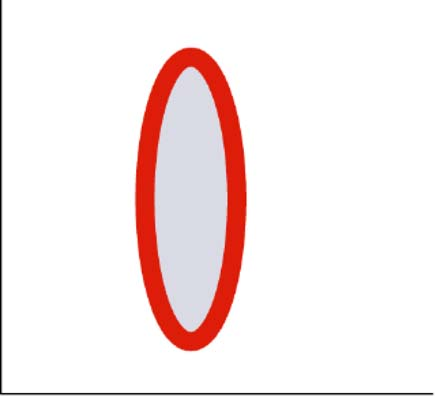
Topographic	Slopes	slope_FL1-SSC_ slope_FL1-FSC_ slope_SSC-FSC_	Absolute slope of type II linear regression	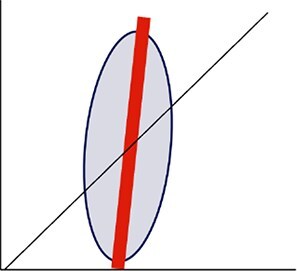
	Centroids of peaks	-	Based on the density function using the SSC-FL1 plane	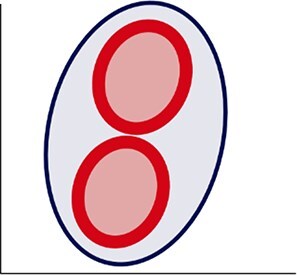
	Cytometric populations	Cytometric populations	Based on the number of peaks identified in the density function	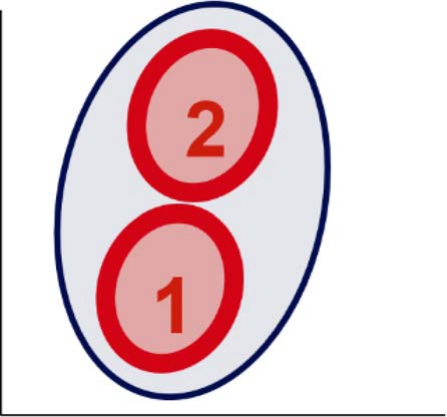

We further calculated two aspects of the topographic dimension of the cytometric dot cloud: the inclination of the ellipsoid, which is based on the slopes of the planes defined by FL1-SSC, FL1-FSC, and SSC-FSC (slope_FL1-SSC_, slope_FL1-FSC_, slope_SSC-FSC_ respectively) using Type II regression major axis method (R package *lmodel2* v.1.7.3) [29]. The distinct cell populations within the cloud were determined using the *k*-means algorithm in the FL1-SSC plane using the R package *flowPeaks* v1.44.0 [30], hosted in Bioconductor v3.16 [31]. The number of density peaks determined was the number of cytometric populations identified, and this latter metric was used to describe the internal heterogeneity of the cytometric dot cloud (Table 1). A *f-test* was computed to check the homogeneity between duplicates. Duplicates that showed a significance level < 0.05, were removed from further analysis. After that, the average of each metric was calculated for the duplicate estimates for the remaining 637 samples. Details for *Step 3: Ellipsoid visualization* and for the maths behind the code used to extract the bacterioplankton cytometric structure are in Supplementary Information.

### Statistical analyses

The metrics that define the BMS and the environmental dataset were normalized (0 to 1) to remove differences in scales prior to multivariate statistical analyses. Prior to the analysis, collinearity (r ≥ 0.6) between the environmental variables and between the metrics was checked using Spearman correlation matrices. To explore the ordination of the lakes based on the environmental dataset, a principal components analysis (PCA) was performed with the Euclidean distance using the prcomp function (R package *stats* version 3.6.2). To explore the ordination of the lakes based on the BMS, a principal coordinate analysis (PCoA) was performed with Bray-Curtis distance using the pcoa function (R package *ape* version 5.8–1). To determine the role and significant of the environmental variables driving the BMS across lakes, a partial least-squares (PLSs) regression analysis was performed with the selection of the 18 environmental variables using the plsr function (R package *pls* version 2.8.5) [32]. All analyses were conducted in R Statistical Software v4 4.1 [33] and RStudio v2024.04.2–764 [34]. Details for statistical analysis are in Supplementary Information.

## Results

### The bacterioplankton morphological structure

**Figure 2:**
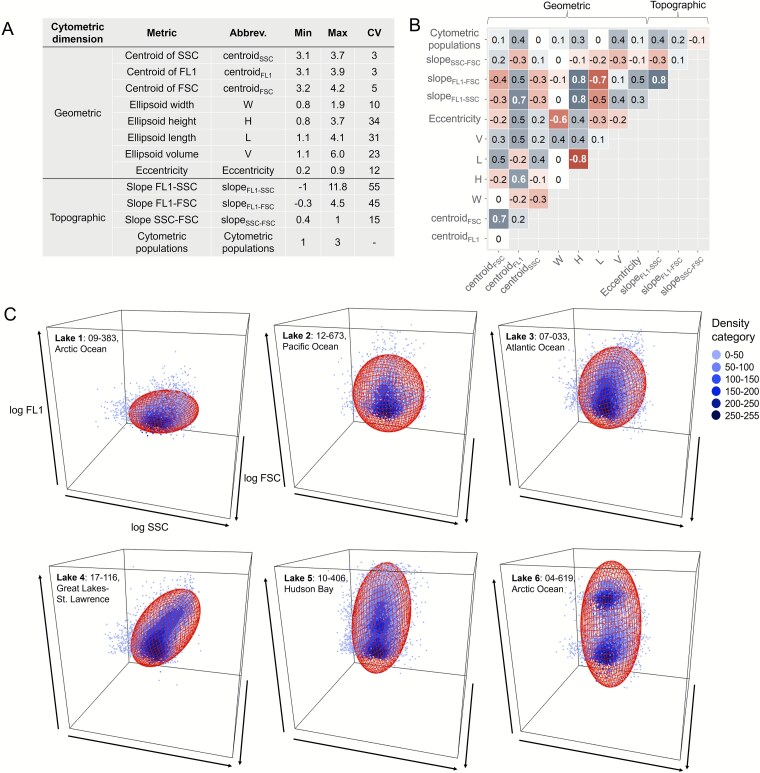
The bacterioplankton morphological structure. (A) Minimum, maximum and coefficient of variation (CV, %) of the cytometric metrics for the entire dataset (*n =* 637), (B) correlation matrix of the cytometric metrics where the degree of correlation is indicated by both the value and the color intensity, and (C) three-dimensional density plots of the bacterioplankton cytometric dot cloud from six random lakes along with their respective ellipsoid at a 90% confidence level. Data points are coloured according to the local density, with lighter blue indicating lower density regions and darker colors corresponding to higher density regions.

Combining the values of SSC, FL1 and FSC allowed us to determine 12 metrics related to the geometric and topographic dimensions of the bacterioplankton cytometric dot clouds across the 637 study lakes. In the geometric dimension, H was the metric that varied the most across lakes (CV 34%), followed by the L (CV 31%) and the V (CV 23%), resulting in a six-fold volume range of ellipsoids across samples (Fig. 2A). Eccentricity varied from 0.2 to 0.9, suggesting that ellipsoids varied from close to a sphere shape (eccentricity ~0) to markedly elongated shapes (eccentricity ~1). In the topographic dimension, slope_FL1-SSC_ and slope_FL1-FSC_ varied in magnitude and sign, from negative to positive values (Fig. 2A). Most of the lakes were composed of bacterioplankton communities with just one cytometric population (*n =* 523 lakes, 82% of the total). A subset of lakes had bacterioplankton communities characterized by two distinct cytometric populations (*n =* 109 lakes, 17% of the total), and only five lakes had three distinct cytometric populations (1%) (Fig. 2A).

On the basis of correlations ≥0.6, and to avoid redundancy in the data (Fig. 2B), we retained seven out of the 12 original cytometric metrics, i.e. H, W, V, centroid_SSC_, slope_SSC-FSC_ and cytometric populations for all subsequent analyses. Note that although eccentricity was not retained for subsequent analysis, a comparison between H and W combined with eccentricity allowed us to categorize the cytometric dot clouds into *prolate* (H > W) and *oblate* (W > H) shapes with different degrees of elongation depending on eccentricity (from 0 to 1), and we used this classification in further analyses. Examples of characteristic ellipsoid shapes are shown in Fig. 2C.

### Environmental gradients across lakes

**Figure 3:**
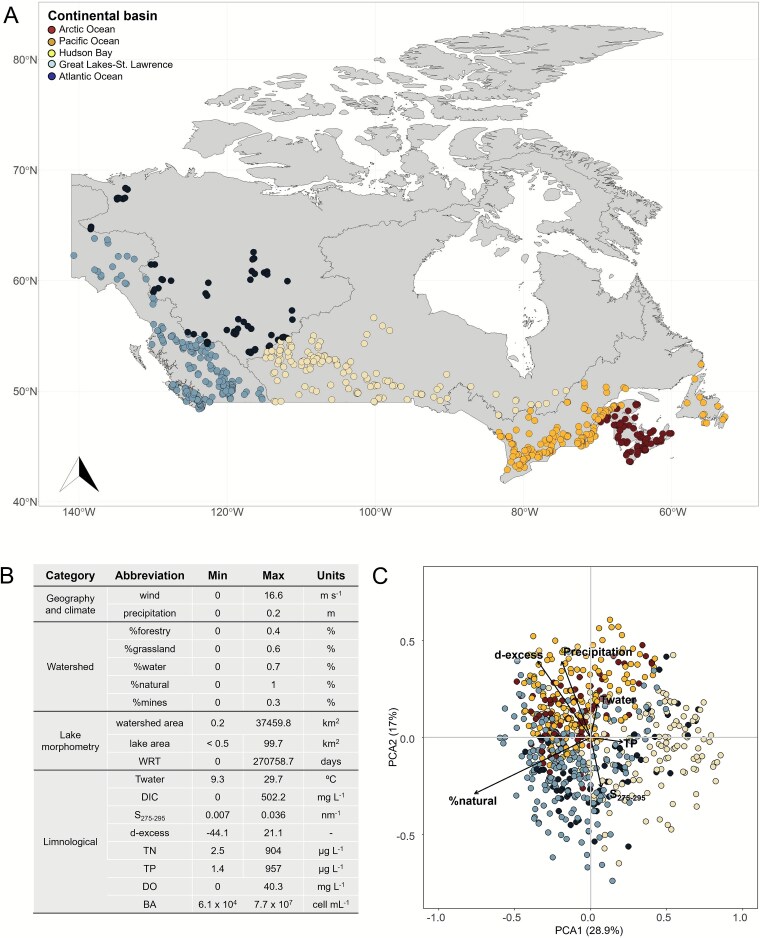
Lake ordination based on the environmental dataset. (A) Map of Canada with samples (*n =* 637) taken in 2017, 2018, and 2019. Colors indicate the continental basins of Canada. (B) a total of 18 environmental variables selected after collinearity, i.e. wind, precipitation recorder over 30 days (precipitation), watershed fraction cover by forest (%forestry), grassland (%grassland), water (%water), natural landscape (%natural), mines (%mines), watershed area, lake area, water residence time (WRT), surface water temperature (Twater), dissolved inorganic carbon (DIC), spectral slope of coloured dissolved organic matter between 275 and 295 nm (S_275–295_), deuterium excess (d-excess), total nitrogen (TN), total phosphorus (TP), dissolved oxygen (DO) and total bacterial abundance (BA), and (C) PCA analysis based on the 18 selected variables. Data points are coloured by the continental basins, and only variables that contributed >5% in both axes have been represented.

A total of 18 environmental variables were retained after checking for collinearity and grouped in the four categories described in the Methods section. Details for the 18 environmental variables are in Fig. 3B and for the full environmental dataset are in Supplementary Table 1.

PCA analysis based on the 18 selected environmental variables explained 45.9% of the total variance of the lakes along the first two axes (Fig. 3C). The lakes tended to group by continental basin (Permanova_Continental basin_: R^2^ = 0.19, *P* = .001) albeit with a large degree of overlap. To simplify interpretation, only variables that contributed >5% at least in one of the two main axes have been represented (Fig. 3C). Lakes were segregated along the continental basins based on climatic differences (precipitation), watershed (%natural), hydrology (d-excess), CDOM source composition (S_275–295_), Twater and trophic status (TP). Briefly, sites varied along a gradient from lakes surrounded by watersheds with a significant %natural located in the continental basins of the Arctic and Pacific Ocean (lower left quadrant) (Fig. 3C) to lakes with higher precipitation and higher d-excess values, located in the Atlantic Ocean and Great Lakes-St. Lawrence regions (upper quadrants) (Fig. 3C). Lakes in the Hudson Bay basin were both the most segregated and the most spread, with higher trophic status (TP) on average (right quadrants) (Fig. 3C).

D-excess emerged as the single most important variable explaining the variability among lakes, and we further assessed the links of d-excess to other key variables (Fig. S1). Note that these trends include variables such as altitude and DOC, which were not incorporated into further analysis due to the string collinearity with d-excess (r = −0.7) and TP (r = 0.7) respectively. Briefly, d-excess was associated to a combination of climatic (precipitation), and watershed features (altitude) and lake features (WRT, TP, DOC). In general, lakes with low d-excess values (high degree of evaporation) had higher WRT and had higher TP and DOC concentrations compared to the lakes with higher d-excess values (low degree of evaporation) (Fig. S1).

### Large scale patterns of the bacterioplankton morphological structure

**Figure 4:**
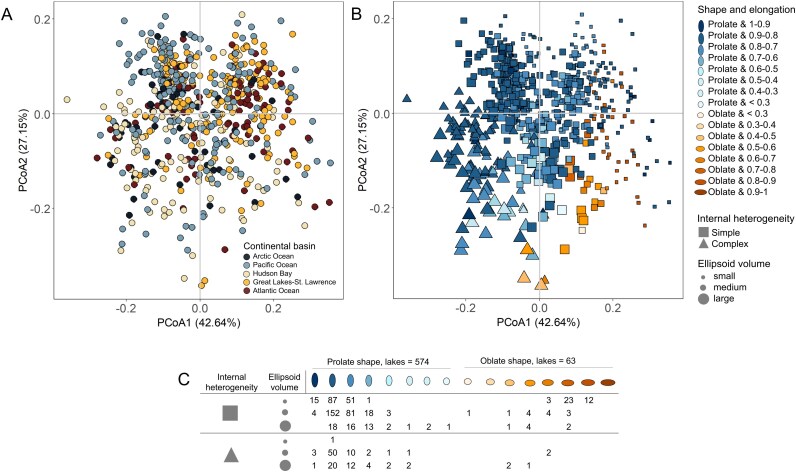
Lake ordination based on the bacterioplankton morphological structure. (A) PCoA analysis based on the seven selected cytometric metrics after collinearity. Data points are coloured by the continental basins, (B) PCoA analysis with data points coloured by shape and elongation category, which is based on the ellipse shape (i.e. prolate: H > W or oblate: W > H) and elongation (i.e. range of eccentricity values: 0 to 1); symbolized by the internal heterogeneity category, which is based on the cytometric populations (i.e. simple: 1 cytometric population and complex: 2 or 3 cytometric population), and sized by the ellipsoid volume category, which is based on its range values (i.e. small: <2.5, medium: 2.5–3.5, large: > 3.5) and (C) classification of the lakes by the shape and elongation category (from highly elongated prolate to highly flattened oblate shape), internal heterogeneity (simple or complex) and ellipsoid volume (small, medium and large). Each cell represents the number of lakes corresponding to a specific combination of these categories.

PCoA analysis based on the selected metrics explained 70.4% of the total variance of the lakes along the first two axes (Fig. 4A). Lakes were not distributed by continental basins as clearly as was observed in the environmental ordination (Fig. 3C), showing smaller differences among the continental basins (Permanova_Continental basin_: R^2^ = 0.082, *P* = .001) and with a larger degree of overlap (Fig. 4A). The first axis, however, which explained 43.96% of the total variance, shows that most of the lakes located in the Pacific Ocean basin were segregated in the upper left quadrant, whereas most of the lakes located in the Atlantic Ocean and Great Lakes-St. Lawrence regions segregated in the upper right quadrant. Interestingly, lakes in the Hudson Bay basin had the greatest segregation as was observed in the environmental ordination (lower quadrants) (Figs 3C, 4A).

To better understand the large-scale trends in the BMS, we categorized the lakes by merging several metrics. These included shape (prolate: H > W or oblate: W > H) and elongation (eccentricity values: 0 to 1) as previously mentioned, the internal heterogeneity (based on cytometric populations: simple: 1 population, complex: 2 or 3 populations), and ellipsoid volume (V) (small: < 2.5, medium: 2.5–3.5, large: > 3.5) (Fig. 4B). A clearer pattern emerged, with highly elongated prolate shapes, simple internal heterogeneity and medium V predominant in the upper left quadrant, while less elongated prolate shapes with complex internal heterogeneity and large V shifted towards the lower left quadrant. On the right side, oblate shapes with simple internal heterogeneity and medium V were in the upper right quadrant with mostly simple internal heterogeneity and large V appearing in the lower right quadrant (Fig. 4B). The number of lakes based on this classification are in Fig. 4C, and representative cytograms illustrating the main BMS aspects are provided in Figs S2 and S3.

### Linking the bacterioplankton morphological structure to environmental drivers

**Figure 5:**
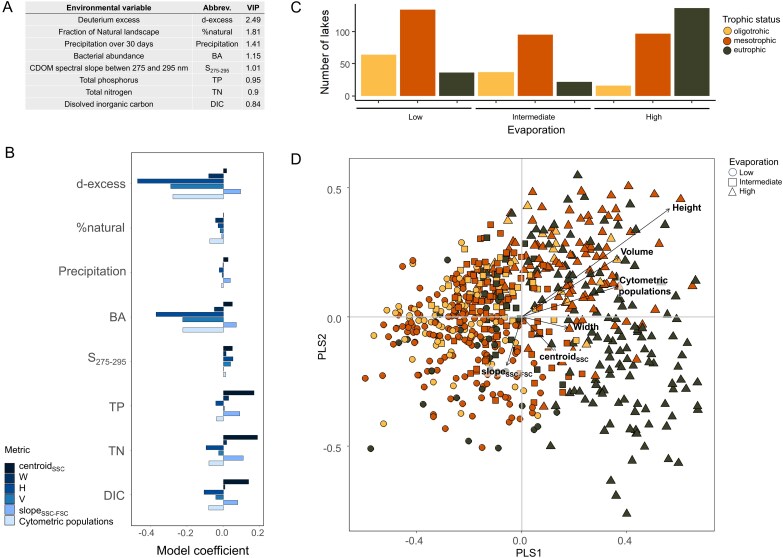
Linking the bacterioplankton morphological structure (BMS) to environmental drivers. (A) the variable importance in projections (VIP) values extracted from the partial least-squares (PLS) regression analysis in a decreasing order. Higher VIP values indicate a greater importance in determining the BMS across lakes. Only variables with VIP ≥ 0.8 have been considered for the PLS analysis, (B) PLS standardized coefficients showing the directions and magnitudes of the effects of each variable on each metric, (C) lakes binned by the combined category of degree of evaporation (low, intermediate, and high) based on the d-excess values: > 0, 0 – −10 and <−10 respectively, and trophic status (oligotrophic, mesotrophic, and eutrophic), based on the TP values: <10, 10–35, and > 35 μg /L respectively, and (D) the first two PLS components based on the selected cytometric metrics and the selected environmental variables. The black labels and arrows represent the selected metrics. Data points are coloured by trophic status, and the symbols represent degree of evaporation.

Links between the BMS and environmental drivers across lakes were assessed through a PLSs regression analysis. The PLS analysis explained 56.92% of the total variation of the BMS considering the 18 selected environmental variables as potential predictors. D-excess had the strongest variable importance in projections value (Fig. 5A), suggesting a strong influence of hydrology on the BMS across lakes. D-excess had a strong negative correlation to H, followed by V and cytometric populations (Fig. 5B). Bacterial abundance was also negatively correlated to H, V and cytometric population (Fig. 5B). Interestingly, TP, TN and DIC were all positively correlated with the metrics related to the variability of cell size i.e. centroid_SSC_, and to a lesser extent W (Fig. 5B). Although %natural, precipitation and S_275–295_ emerged as having a strong variable importance in projections value, they did not strongly correlate with any of the cytometric variables individually, suggesting that watershed features in combination to precipitation and CDOM properties may influence the ensemble of the metrics that determine the BMS across lakes (Fig. 5A and B).

Based on the model coefficient with the individual metrics and their orthogonality in the first two PLS components representation (Fig. 5A and B and Fig. S4), we binned the lakes into categories of d-excess (> 0, 0 – −10 and < −10) and TP (< 4 10, 10–35 and > 35 μg/L following thresholds for Canadian freshwater systems). This allowed us to classify the lakes based on the degree of water evaporation (low, intermediate and high) and trophic status (oligotrophic, mesotrophic, and eutrophic) (Fig. 5C). The first two PLS components showed a clear distribution of lakes characterized by almost orthogonal gradients of the degree of evaporation and trophic status as previously mentioned (Fig. 5D, Fig. S4). Although the pattern was less pronounced in intermediate trophic conditions, most lakes with low degree of evaporation and oligotrophic conditions were located in the upper left quadrant. In contrast, lakes with a high degree of evaporation and eutrophic status were mostly located in the lower right quadrant (Fig. 5D).

**Figure 6:**
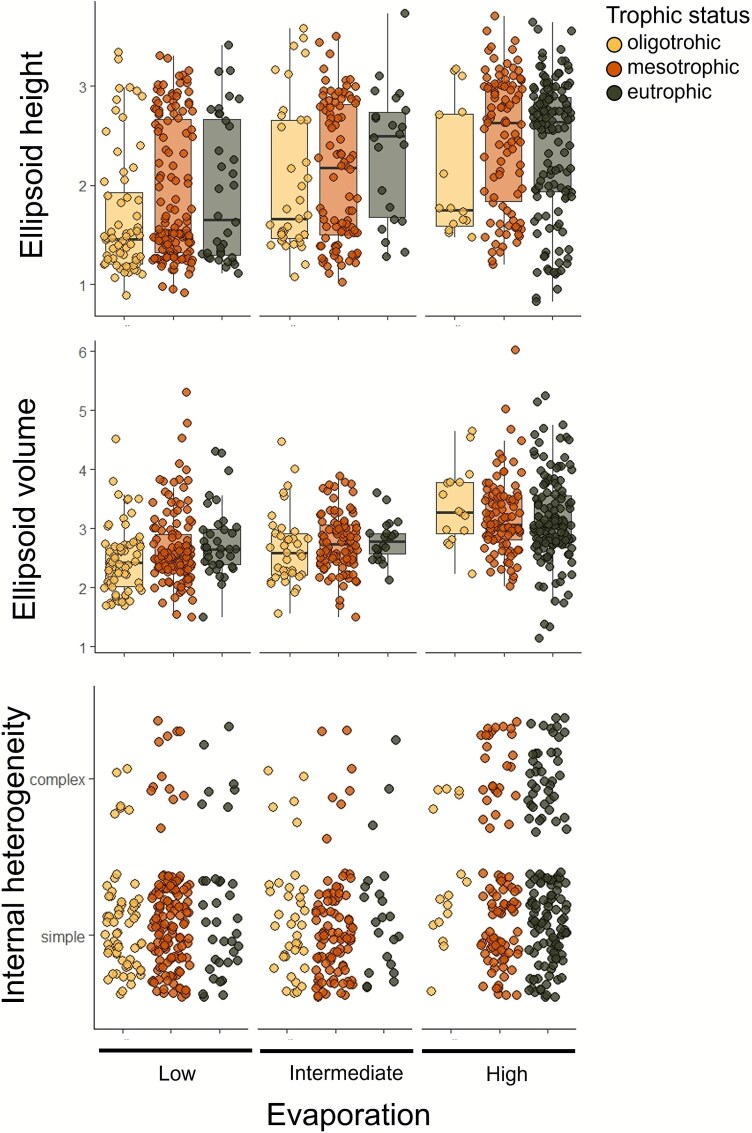
Patterns of ellipsoid height, ellipsoid volume and cytometric populations of the bacterioplankton morphological structure along hydrologic and trophic status gradients. Lakes are grouped by degree of water evaporation (low, intermediate, high), with trophic status (i.e. oligotrophic, mesotrophic, and eutrophic) nested within each category. Boxplots display the 10th, 25th, 50th (median), 75th and 90th percentiles. Data points are coloured by trophic status.

To further explore large-scale trends in the BMS, we classified individual metrics along gradients of both increasing evaporation and nutrient enrichment (Fig. 6). This exercise shows that along these gradients, there was an increase in the H, V as well an increase of complex internal heterogeneity in eutrophic lakes with high degree of evaporation. Interestingly, eutrophic lakes with a high degree of evaporation showed the widest diversity of H and V (Fig. 6A and B). It is important to highlight that 44% of the lakes with large V had complex internal heterogeneity (Fig. S5). Repeating the same exercise using WRT, we observed that there was an increase of H and V in eutrophic lakes regardless of their WRT (Fig. S6).

## Discussion

The cytometric parameters of the dot cloud allow to characterize what we have termed as the “morphological structure” of the bacterioplankton community, which represents a physical fingerprint, and it is unique to each sample or community. The BMS is analogous to other fingerprint methods that have been developed to characterize and describe different aspects of microbial communities, which include genomic approaches based on environmental DNA [35, 36], enzymatic approaches and substrate consumption profiles [37, 38] and functional approaches [39, 40]. In this regard, the BMS is the phenotypic expression of the taxonomic and phylogenetic structure of the community and of the ensemble of functional traits associated with this.

The BMS proposed in this study captures the different dimensions of the cytometric dot clouds generated by the ensemble of bacterioplankton cells in a sample, in terms of geometry, describing the shape, elongation and volume of the cytometric dot cloud, and in terms of topography, defined by the orientation and internal heterogeneity of the cytometric dot cloud. Although internal heterogeneity reflects the detected cytometric populations, it is important to note that it can vary depending on the flow cytometer used, since instrument sensitivity differs [41]. Samples analyzed with a more sensitive instrument might therefore show slightly different patterns of the cytometric parameters. However, this technical variation does not compromise the basic BMS framework that we are proposing, which is meant to capture the geometry and topography of the dot clouds of bacterioplankton communities regardless of instrument performance.

**Figure 7:**
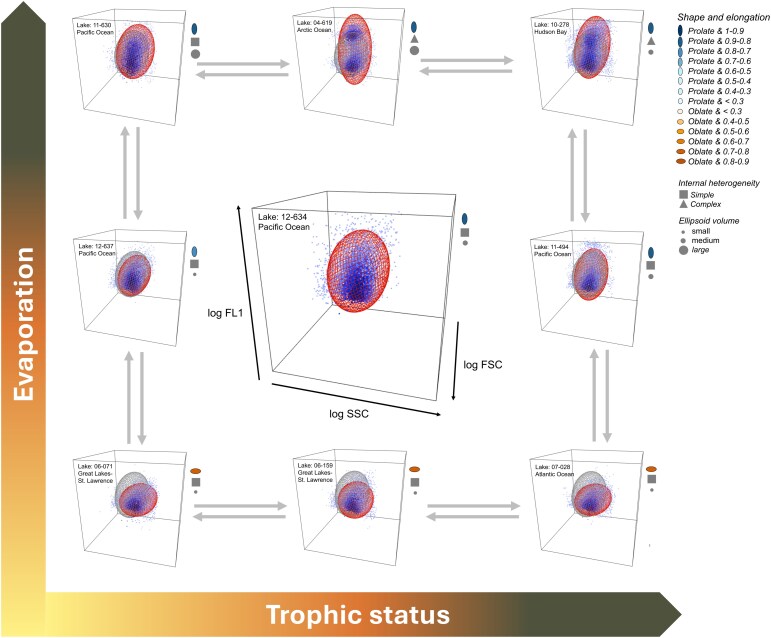
The bacterioplankton morphological structure (BMS) succession along environmental gradients. Conceptual figure showing the gradual changes of the BMS along the key environmental gradients, which were selected by the PLS regression analysis. Notice that the grey arrows between every plot are bidirectional because these gradual changes can be in both ways, including with the central plot. The grey ellipsoid in each plot represents the central ellipsoid (Lake: 12–634) as a reference to easily visualize the changes. The symbols in the right part of each plot correspond to the specific lake trophic status and the shape, elongation, internal heterogeneity and ellipsoid volume of the cytometric dot cloud of the bacterioplankton community for that lake.

We have applied this framework to lake water samples taken in the context of the pan-Canadian LakePulse project, the first standardized national lake survey across 637 lakes that covers wide environmental, climatic and human impact gradients [22]. This likely represents the largest standardized cytometric study of freshwater bacterioplankton ever carried out and allowed us to quantify and analyze in a consistent manner the variability in the BMS across a wide range of lake scenarios. Hydrology and trophic status emerged as the two major factors influencing the BMS across Canadian lakes. We have shown that the changes in the BMS are not random, but rather there is a gradual diversification and complexification of the BMS along these two gradients. This unique community fingerprint therefore varies in consistent and directional ways along key environmental gradients, suggesting that the BMS may be integrating a variety of community responses to environmental shifts (Fig. 7).

### The bacterioplankton morphological structure responds to lake and watershed properties

Our analysis suggests that the variability in the BMS is greatly influenced by hydrology and lake trophic status gradients, and that there is both a diversification and a complexification in the BMS along gradients of both increasing evaporation and nutrient enrichment. D-excess, an isotopic index that integrates water origin and extent of evaporation [42, 43] was the variable most strongly influencing the BMS across lakes. In turn, along with this evaporative gradient, there was an increase in the proportion of eutrophic lakes (Fig. 5C). This pattern reflects in part the lakes in the central Prairies region of the Hudson Bay Basin, and shallow subarctic lakes in Arctic Ocean basin, all of which have very limited hydrologic connectivity and extremely long residence times, and which are in many cases eutrophic due to either watershed properties, or the influence of agriculture in the case of the Prairie lakes. It should be noted that these lakes are often characterized by high concentrations of DOC, which are in part due to the accumulation of mostly photodegraded and highly recalcitrant DOC over long water residence times [44, 45].

D-excess at any given lake is influenced by a combination of climatic, hydrologic and watershed properties, as well as the residence time of water in the lake (Figs S1A–D). This implies that d-excess reflects not just water residence time and internal processes within a given lake, but also the upstream hydrologic history, including the configuration of the aquatic network, the extent of land / water interactions and a variety of landscape features [43, 46]. The stronger association of the BMS to d-excess rather than to WRT (Fig. 6, Fig. S6) implies that the BMS likely reflects not only internal lake processes, but also the network history of the water and of the microbial communities that travel with it. The role of hydrologic connectivity, such as stream inputs, watershed position and WRT, in shaping bacterioplankton community composition and function has been highlighted before [47–50], and here we demonstrate that the influence of hydrology extends to the physical fingerprint of the community.

The nutrient enrichment that occurs along hydrologic gradients was associated to a progressive transition in cytometric dot clouds, where oligotrophic lakes with low degree of evaporation (> d-excess) had bacterioplankton communities that were predominantly oblate shaped, with small ellipsoid volume and simple internal heterogeneity, whereas eutrophic lakes with higher degree of evaporation (< d-excess) had BMS with higher diversity of shapes, larger ellipsoid volume and complex internal heterogeneity (Figs. 6 and 7). In parallel to the nutrient enrichment along hydrologic gradients, gradients of other lake features were also observed. For example, we observed a progressive increase in DOC concentration along the hydrologic gradient (Fig. S1F), accompanied by an increase in low molecular weight (MW) components (> S_275–295_) (Fig. S7), which are often associated with autochthonous sources such as microbial activity, algal exudates and photochemical degradation [28, 51]. In turn, these gradients in DOC concentration and MW components were associated with changes in the BMS that were analogous to the ones observed along the nutrient enrichment gradient (Figs S8 and S9). This suggests that the physical fingerprint of the community may respond not just to nutrient concentrations, but also to varying organic matter in terms of quality and availability.

In lakes in the boreal biome, higher dissolved organic matter (DOM) concentrations have been associated with increased chemodiversity (DOM molecular diversity), with terrestrially derived DOM being selectively lost as WRT increases [52, 53], and Shahabinia *et al.* in press. Moreover, there is a wealth of published evidence suggesting that the origin and quality of DOM plays a role in shaping the taxonomic composition and function of bacterioplankton communities [40, 54, 55]. Although describing the changes of DOM composition and chemodiversity is beyond the scope of this paper, our findings would suggest that changes in DOM composition along hydrologic gradients may further influence the BMS across lakes. Regardless, our results suggest that the BMS responds to key environmental gradients and that this structure reflects water history, connectivity to land, availability of nutrients, and dissolved organic matter sources and composition, and possibly other internal lake processes.

### Ecological significance of the bacterioplankton morphological structure based on cytometric parameters

It has been well established that taxonomic successions within microbial communities occur along environmental gradients, and in particular, along the aquatic continuum from headwater streams, which are tightly connected to the surrounding soils, to larger rivers and lakes with longer residence times [50, 56–58]. It has been shown that in habitats with short WRT, microbial communities tend to resemble those of upstream waters and of the surrounding catchment [40, 57]. In contrast, systems with longer WRT experience increased local selection processes, leading to the establishment of taxa within the microbial communities that are favored by the prevailing environmental conditions. In this context, one possible interpretation of our results is that the shifts in the BMS that we observed represent the physical / phenotypic expression of the taxonomic succession i.e. occurring along these gradients, driven by a combination of mass effects and species sorting and selection [59]. In this regard, the BMS shifts from simpler and smaller clouds in lakes with less evaporated water (and lower overall WRT), which likely represent lake communities that are dominated by mass effects and populated mostly by allochthonous taxa, to larger, more elongated and more dispersed clouds, ultimately developing several distinct cytometric populations in lakes with more evaporated water and longer WRT, as local selection leads to a numerical dominance of predominantly aquatic bacteria, which may have a very distinct cytometric signature [60, 61].

These observations also lead us to redefine the concept of High- and Low-DNA fractions, which have been reported across both freshwater and marine ecosystems [62–65] among many others. In our study, however, this configuration of coexisting High and Low-DNA fractions was not the prevailing structure observed in most lakes. Rather, there was a continuous shift in the shape of the dot clouds in terms of elongation, volume and complexity along hydrologic and trophic gradients, with the presence of two distinct fractions being the endpoint of this succession in community BMS across lakes, expressed only under certain environmental scenarios. In this revised framework, the Low-DNA fraction represents a core BMS i.e. characterized by small, simple and oblate shapes in addition to low overall fluorescence. We hypothesize that this core is mainly populated by taxa linked to the rare portion of the community, likely a combination of allochthonous taxa that are largely inactive, and autochthonous taxa that may have become maladapted to the local environment. This core structure is present in all communities, including oceans, but in lakes it is dominant in communities most strongly influenced by mass effects and external inputs of taxa, and the most extreme example is the bottom left structure in Fig. 7, from a lake that has very low evaporated water and low WRT. As environmental selection becomes more prevalent along gradients of increasing network and lake water residence time and system productivity, a mounting number of taxa are outside this core, increasingly diverging in cytometric properties due their higher growth rates reflected in higher DNA / RNA signals, and also in their wider range in size and morphology. This drives the shifts in BMS that we observed along gradients and that are exemplified in the shifting configurations in Fig. 7. In this framework, the core is not only present in all samples and communities regardless of lake conditions, but its cytometric properties remain essentially unchanged among communities, determining a baseline structure across lakes and communities that anchors the base of the ellipsoid. The succession in community BMS that occurs along environmental gradients therefore does not involve shifts in the core itself (or what has been referred to as low-DNA fraction), but rather the progressive emergence of cells with higher DNA contents and with more diverse cell morphometry. This results in an overall elongation and increase in volume (i.e. dispersion) and complexity of the structure, which culminates in community morphological structures that have 2 (sometimes 3 or more) distinct cytometric populations, as shown in the upper right structures in Fig. 7. The emergence of a second distinct cytometric population, which likely corresponds to the so-called high-DNA fractions, is probably associated with communities characterized by steeper rank abundance curves with more marked dominance by a small number of dominant taxa. This represents the endpoint of a continuous BMS succession that occurs along a gradient of increasing WRT within the aquatic network, as reflected in d-excess, and also a gradient of system productivity, and therefore of increasing resource availability.

The above interpretation is not incompatible with the recurrent assumption that high and low-DNA fractions represent bacterial cells with high and low activity or growth, respectively [66–68], but places this assumption in the context of a continuous succession pattern along a gradient of network WRT, productivity and diversification of resources. These fractions are prevalent in marine samples as well [62, 69, 70], and there the BMS succession may occur not along a gradient of water residence time within water masses, but rather along gradients of productivity in upwelling areas e.g. The concept of a continuous progression from a basic expression of the cytometric dot cloud, characterized by flatter, smaller and simpler structures, which tends to complexify along various environmental gradients, resulting in a structural divergence of bacterioplankton communities likely applies to marine habitats as well. In this regard, it is important to note that this progression was not always clear along a single environmental gradient, suggesting that the morphological structure of the bacterioplankton community is influenced by multiple interacting factors, including other bacterial fingerprints, and not solely determined by gradients considered here. Further studies, therefore, are needed to understand how this BMS succession is linked to shifts in bacterial composition and function, and how these links are established, i.e. whether the BMS is a result of the taxonomic structure, which itself is shaped by environmental factors, or whether the environment is acting directly on the physical fingerprint of the community, and this then trickles down to impact the taxonomic and functional dimensions of the community.

## Supplementary Material

A_cytometric_framework_suppl_info_FINAL_version_ycaf223

## Data Availability

Data used in this study are deposited in the *metaGRIL* hosted on Borealis, the Canadian Dataverse Repository, doi:http://dx.doi.org/10.5683/SP3/S8TACN. The code for the cytometric data extraction is available on Github https://github.com/sara-soriap/Paper_Soria-Piriz_delGiorgio_ISMEComm_2025.
