# Supplementary material for: A cytometric framework to assess trends in the morphological structure of bacterioplankton communities along freshwater environmental gradients
Source: ISME Commun. 2025 Nov 25;5(1):ycaf223. doi: 10.1093/ismeco/ycaf223 (PMC12694418; doi:10.1093/ismeco/ycaf223)
Supplement: A_cytometric_framework_suppl_info_FINAL_version_ycaf223 [file a_cytometric_framework_suppl_info_final_version_ycaf223.docx]

**Supplementary information**

**A cytometric framework to assess trends in the morphological structure of bacterioplankton communities along freshwater environmental gradients**

**Sara Soria-Píriz**^1^***, Paul A. del Giorgio**^1^

^1*^Carbon Biogeochemistry of Boreal Aquatic Systems (CarBBAS) group, Groupe de Recherche Interuniversitaire en Limnologie (GRIL), Département des Sciences Biologiques, Université du Québec à Montréal, Montréal, Québec, Canada.

* Université du Québec à Montréal, Pavillon des sciences biologiques, SB-2855, 2080 St-Urbain, Montréal, Québec, H2X 3X8, Canada

email: [soria_piriz.sara@uqam.ca](mailto:soria_piriz.sara@uqam.ca)

**Material and Methods**

**Environmental dataset**

*Limnological* category included chemical variables such as surface water temperature (Twater, ºC), pH, specific conductivity, salinity and dissolved oxygen (DO, mg/mL) which were performed on site (field protocols described in NSERC Canadian Lake Pulse Network, 2021). The ions concentrations of magnesium, potassium, calcium, sodium and chloride were determined following U.S. Environmental Protection Agency protocols (1994, 1997). Samples for δ^2^H and oxygen δ^18^O_2_ of ambient surface water were collected in 30 mL HDPE bottles without any air bubbles, and analyzed using an LGR (Los Gatos Research) model T-LWIA-45-EP Off-Axis Integrated Cavity Output Spectroscopy (OA-ICOS). Deuterium excess (d-excess) values were calculated as follows: d-excess = δ^2^H - 8 * δ^18^O_2_ [1]. d-excess represents a departure from the local meteoric signal and represents an index of the degree of evaporation in a given water sample. Dissolved organic and dissolved inorganic carbon (DOC and DIC respectively, mg/L) measurements are described in [2]. Methodology for determining chlorophyll *a* concentration (Chl *a,* µg/L), absorption of non-algal particles at 443 nm (PABS_443_, m^-1^) are provided in [3]. Total suspended solids (TSS, mg/L), organic suspended particulate matter (OSPM, mg/L) and mineral suspended particulate matter (MSPM) concentration were determined following the established protocols by Environmental and Climate Change Canada. Further methodological details about TSS and OSPM can be found in [3]. Total phosphorus (TP, µg/L) was analyzed according to [4]. Total nitrogen (TN, µg/L) was determined following [5]. The spectral slope of the coloured dissolved organic matter (CDOM) was calculated for the wavelength interval of 275 to 295 nm (S_275-295_) following [6]. S_275-295_ represents a proxy of the CDOM molecular weight (MW), and source composition indicating a high spectral slope, a major proportion of low MW, typically derived from recently produced organic matter. In contrast, a lower spectral slope suggests a greater proportion of high MW, which is often associated with degradation processes (Helms et al. 2008). Determining the bacterial cell abundance (BA) is described below.

**Flow cytometric analysis**

Samples were left to thaw at room temperature, 400 µL aliquots were stained with SYTO^TM^ 13 (Molecular Probes #S7575) (2.5 µM final concentration) and incubated for 10 min at room temperature in darkness. Stained bacterial cells were excited at 488 nm and detected according to their 90° light side scatter (SSC, related to the granularity and complexity of the cells), forward scatter (FSC, related to the cell size) and green fluorescence (FL1, used as a proxy of nucleic acid cellular content) [7–9]. The average of the bacterial cell abundance (BA) was calculated for each pair of duplicates with a coefficient of variation < 10% (C.V., i.e., ratio between the standard deviation and average of the duplicate values). Samples were processed again when the C.V. > 10%. Standard *.fcs* files were extracted for later data processing. The method described above is based on that published by [10]. The BA in each sample was subsequently determined, based on the number of events within the common gate (*Step 1: Cytometric data preparation* in the main manuscript), the volume of the sample analyzed and the dilution factor (if applicable). An average of the BA was calculated between duplicates for the 637 samples. BA was not considered as part of the bacterioplankton morphological structure (BMS) but included in the environmental dataset.

**Defining the bacterioplankton cytometric structure**

**Step 3: Ellipsoid visualization**

A three-dimensional projection using the values of the SSC, FL1 and FSC at log scale was carried out to visualize the cytometric dot cloud as a density dot plot and the 90% covariance error ellipsoid (Fig. 2). The densCols() function was used to extract the kernel density of each dot using a gaussian distribution. Fixed values of each argument of the function (i.e., *nbin,* *bandwidth* and *colramp)* were applied to be consistent among duplicates and samples. (R package grDevices v 3.6.2). The col2rgb() function was used to convert the HEX code output of the densCols() (ex. "#A5A5A5") in RGB decimal (i.e. from 0 to 255) of every dot (R package grDevices v 3.6.2). The RGB decimal output was classified in six categories in an increasing density as well as color (i.e. 0-50, 50-100, 100-150, 150-200, 200-250 and 200-250) (Fig. 2). The plot3d() function was used to visualize the 3-dimensional density dot plot and the ellipse3d() function was used to visualize the ellipsoid, whose arguments are the centroid, the correlation matrix and the standard deviation of the values of SSC, FL1 and FSC parameters for a confidence level of 90%. The wire3d() function was used to define the mesh of the ellipsoid (R package rgl v1.2.1) [11]. All analyses were conducted in R Statistical Software v4 4.1 [12] and RStudio v2024.04.2-764 [13].

**The mathematics behind the code used to extract the bacterioplankton cytometric structure**

**Lengths of an ellipsoid**

**Step 1 - Determine a covariance matrix for 3D data:**

$$\Sigma=(Var\left( X \right) Cov\left( Y,X \right) Cov(Z,X)$$

$$Cov\left( X,Y \right) Var\left( Y \right) Cov(Z,Y)$$

$$Cov\left( X,Z \right) Cov\left( Y,Z \right) Var(Z))$$

This matrix summarizes how wide the data is in each direction (variances on the diagonal) and how tilted the cloud is (covariances off diagonal).

In R, this is simply:

covMat <- cov(data3D),

**Step 2 - Determine the eigenvalues and eigenvectors of the ellipsoid**

With the covariance matrix determined previously, we compute the **eigenvectors**, which define the directions of the axes in the 3D data cloud, and the **eigenvalues**, which indicate how much variance there is along each axis (i.e. how stretched or compressed the axis is).

There is no need to manually solve the determinant or cubic equation; R performs this internally:

evals <- eigen(covMat)$values

evecs <- eigen(covMat)$vectors

R sorts the eigenvalues from largest to smallest:

$$\lambda_{1}{{>\lambda}_{2}>\lambda}_{3}$$

This ranking, however, does **not** correspond directly to SSC, FL1 or FSC, because the ellipsoid axes are rotated combinations of the original variables. Therefore, an additional step is required before determining the ellipsoid lengths.

**Step 3 – Assign eigenvalues to cytometric parameters**

To associate each eigenvalue with the correct cytometric parameter, we use the standard deviation (sd) of SSC, FL1 and FSC. The variable with the largest sd contributes most to the largest eigenvalue (i.e., the longest ellipsoid axis), the second largest sd to the second eigenvalue, and so on:

For example, if in a given lake:

FSC_sd_ > FL1_sd_ > SSC_sd_,

then the eigenvalues for that lake are assigned as:

$$\lambda_{1}= \lambda_{FSC}, \lambda_{2}= \lambda_{FL1}, \lambda_{3} =\lambda_{SSC}$$

**Step 4 - Determine the ellipsoid lengths (90% confidence):**

A confidence ellipsoid is defined by:

$$radiusᵢ = \sqrt{\chi^{2}₍₃,₀.₉₀₎ \cdot\lambdaᵢ}$$

where radius_i_ is the distance from the center to the surface along the eigenvector *i.* For 3 degrees of freedom and 90% confidence:

$$\chi^{2}₍₃,₀.₉₀₎=6.251$$

Thus, each axis length is:

$$Lengthᵢ = 2 \times\sqrt{6.251 \times\lambdaᵢ}$$

*Note: We multiple by 2 because the axis length (the full ellipsoid axis) is twice the radius, i.e.,* Length_i_ = 2 x radius_i_

In our case, the ellipsoid lengths are:

Width (W), calculated in the SSC axis: $W=2\times\sqrt{6.251\times\lambda_{SSC}}$

Height (H), calculated in the FL1 axis: $H= 2\times\sqrt{6.251\times\lambda_{FL1}}$

Length (L), calculated in the FSC axis: $L = 2\times\sqrt{6.251\times\lambda_{FSC}}$

**Ellipsoid volume**

Ellipsoid volume (V) was calculated using the standard formula:

$$V = 4/3 \pi abc$$

where *a*, *b*, and *c* are the ellipsoid semi-axis (i.e., half of W, H, and L respectively)

.

***Eccentricity***

The *eccentricity* $(e)$, an index that describes the elongation of an ellipse, was calculated using the standard formula:

$$e=\sqrt{1-\frac{b^{2}}{a^{2}}}$$

where *a is* the semi-major axis, *b* is the semi-minor axis and *e* is the resulting eccentricity of the ellipse.

**Note**: Since width (W) and height (H) may vary between lakes, with W > H in some cases and H > W in others, the assignment of the semi-major (*a*) and semi-minor (*b*) axes depends on which dimension is greater for each lake:

If W > H, then *a* = W/2 and *b* = H/2

If H > W, then *a* = H/2 and *b* = W/2

**Statistical analysis**

The absorption spectra of each sample was fitted to a single exponential decay function using cdom_fit_exponentail function (R package *cdom* version 0.1.0) [14]. Prior to analysis, for the Limnological category variables, a chi-squared test was carried out to check for outliers using the chisq.out.test function (R package *outliers* version 0.15) [15]. Outlier values were replaced by the mean. For the same Limnological category, missing values were filled using the missForest function (R package *missForest* version 1.5) [16]. Geographical differences (i.e. Continental basins) in both environmental variables and the BMS were tested using Permutational analysis of variance (PERMANOVA) on the Euclidean and Bray-Curtis distance matrices respectively (R package *vegan* version 2.5-6) [17]. Prior to the partial least-squares analysis, a cross-validation of components was checked using the root mean squared error method. The relative importance of the environmental variables was determined with the values of variable importance for projection (VIP) using the VIP function (R package *plsVarSe*l version 0.9.12) [18]. The variables with VIP < 0.8 were not represented.

**References**

1. Dansgaard W. Stable isotopes in precipitation. *Tellus* 1964;**16**:436–68, DOI: 10.1111/j.2153-3490.1964.tb00181.x.

2. Kraemer SA, Barbosa Da Costa N, Shapiro BJ, Fradette M, Huot Y, Walsh DA. A large-scale assessment of lakes reveals a pervasive signal of land use on bacterial communities. *The ISME Journal* 2020;**14**:3011–23, DOI: 10.1038/s41396-020-0733-0.

3. Cremella B, Bélanger S, Huot Y. Aquatic particulate absorption coefficient combining extraction and bleaching optimized for inland waters. *Limnology and Oceanography: Methods* 2022;**20**:451–65.

4. Wetzel RG, Likens G. *Limnological Analyses*. Springer Science & Business Media, 2000.

5. Patton CJ, Kryskalla JR. *Methods of Analysis by the US Geological Survey National Water Quality Laboratory: Evaluation of Alkaline Persulfate Digestion as an Alternative to Kjeldahl Digestion for Determination of Total and Dissolved Nitrogen and Phosphorus in Water*. US Department of the Interior, US Geological Survey, 2003.

6. Helms JR, Stubbins A, Ritchie JD, Minor EC, Kieber DJ, Mopper K. Absorption spectral slopes and slope ratios as indicators of molecular weight, source, and photobleaching of chromophoric dissolved organic matter. *Limnology & Oceanography* 2008;**53**:955–69, DOI: 10.4319/lo.2008.53.3.0955.

7. Gasol JM, Zweifel UL, Peters F, Jed A, Zweifel ULI, Fuhrman JEDA. Significance of Size and Nucleic Acid Content Heterogeneity as Measured by Flow Cytometry in Natural Planktonic Bacteria. *Applied and Environmental Microbiology* 1999;**65**:4475–83.

8. Lebaron P, Servais P, Baudoux A, Bourrain M, Courties C, Parthuisot N. Variations of bacterial-specific activity with cell size and nucleic acid content assessed by flow cytometry. *Aquatic Microbial Ecology* 2002;**28**:131–40, DOI: 10.3354/ame028131.

9. Morán XAG, Bode A, Suárez LÁ, Nogueira E. Assessing the relevance of nucleic acid content as an indicator of marine bacterial activity. *Aquatic Microbial Ecology* 2007;**46**:141–52, DOI: 10.3354/ame046141.

10. del Giorgio PA, Bird DF, Prairie YT, Planas D. Flow cytometric determination of bacterial abundance in lake plankton with the green nucleic acid stain SYTO 13. *Limnology and Oceanography* 1996;**41**:783–9, DOI: 10.4319/lo.1996.41.4.0783.

11. Murdoch D, Adler D. rgl: 3D visualization using OpenGL. 2022.

12. R Core Team: A language and environment for statistical computing. 2024.

13. RStudio Team. RStudio: Integrated Development Environment for R. 2024.

14. Massicotte P, Markager S. Using a Gaussian decomposition approach to model absorption spectra of chromophoric dissolved organic matter. *Marine Chemistry* 2016;**180**:24–32, DOI: https://doi.org/10.1016/j.marchem.2016.01.008.

15. Komsta L, Komsta ML. Package ‘outliers.’ *Medical University of Lublin, Lublin* 2011;**879**.

16. Stekhoven DJ, Stekhoven MDJ. Package ‘missForest.’ *R package version* 2013;**1**:21.

17. Oksanen J, Blanchet FG, Kindt R, Legendre P, Minchin PR, O’hara RB *et al.* Package ‘vegan.’ *Community ecology package, version* 2013;**2**:1–295.

18. Mehmood T, Liland KH, Snipen L, Sæbø S. A review of variable selection methods in partial least squares regression. *Chemometrics and intelligent laboratory systems* 2012;**118**:62–9.

**Table S1:** Dataset of the environmental variables classified by category i.e. *Geography and climate*, *watershed*, *lake morphometry* and *limnological*.


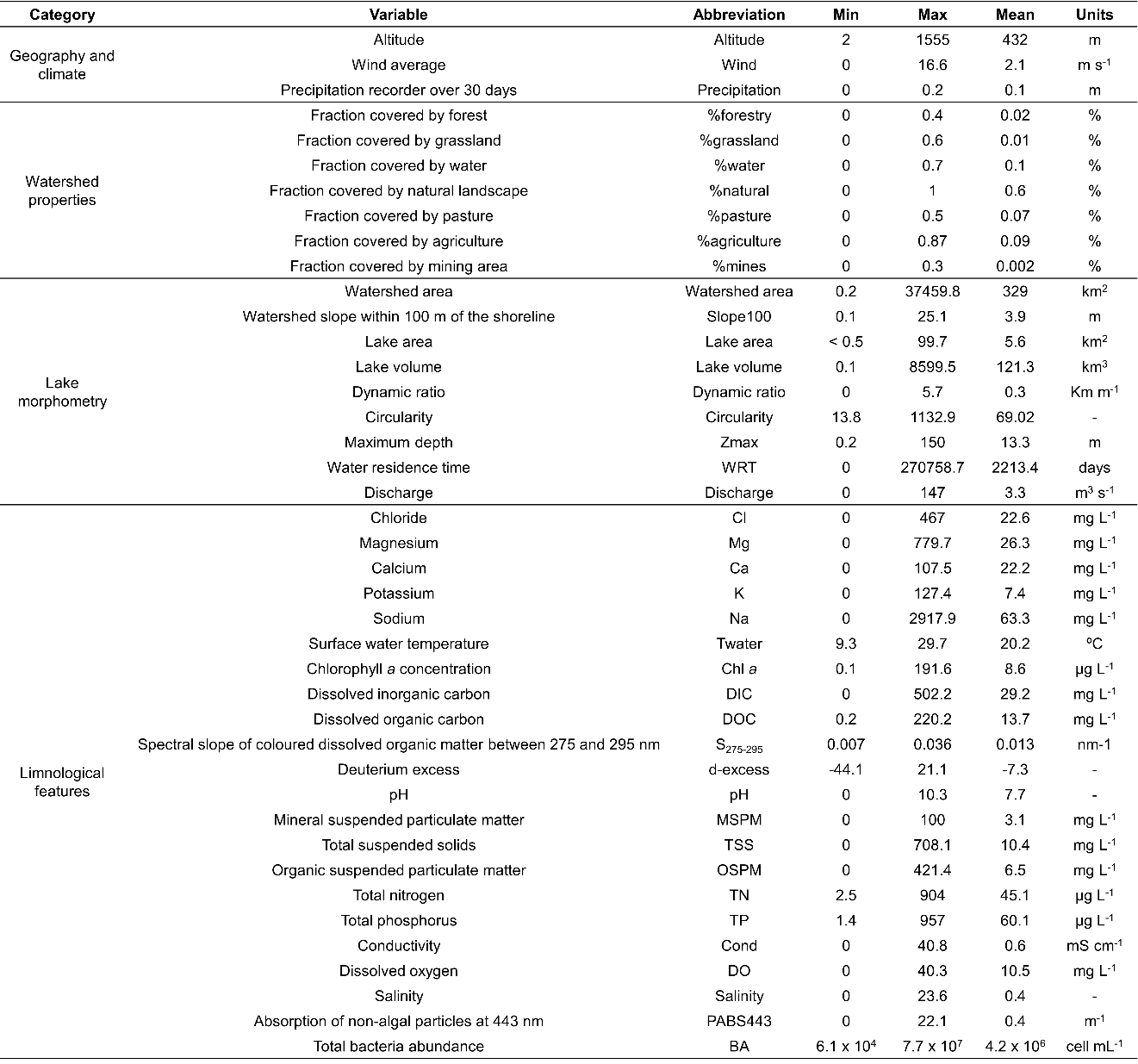


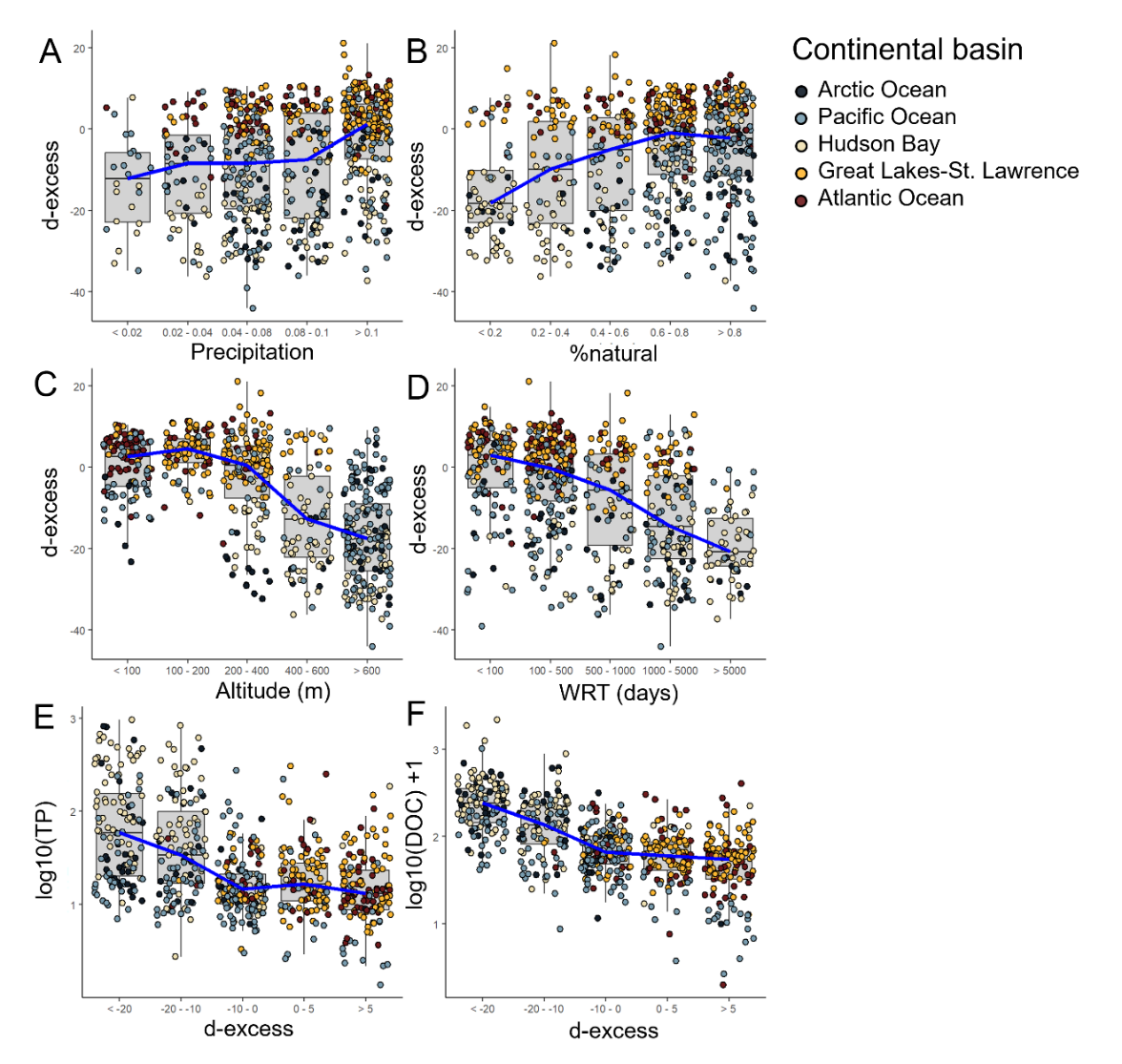


**Figure S1**: **Patterns of environmental variables.** Box plots showing the distribution of the lakes based on categories of **A**) precipitation vs d-excess values, **B)** watershed fraction cover by natural landscape (%natural) vs d-excess values, **C)** altitude (m) vs d-excess values, **D)** water retention time (WRT) versus d-excess values, **E)** d-excess values vs total phosphorus (TP) concentration in log_10_ scale and **F)** d-excess values vs dissolved organic carbon (DOC) concentration in log_10_ scale + 1. Lower to upper values are indicated, respectively, 10%, 25%, 50% (median), 75% and 90%. Data points are coloured by the continental basins. Note that in panels E and F, d-excess is used as an independent variable to assess how TP and DOC concentrations are influenced by the degree of lake evaporation.


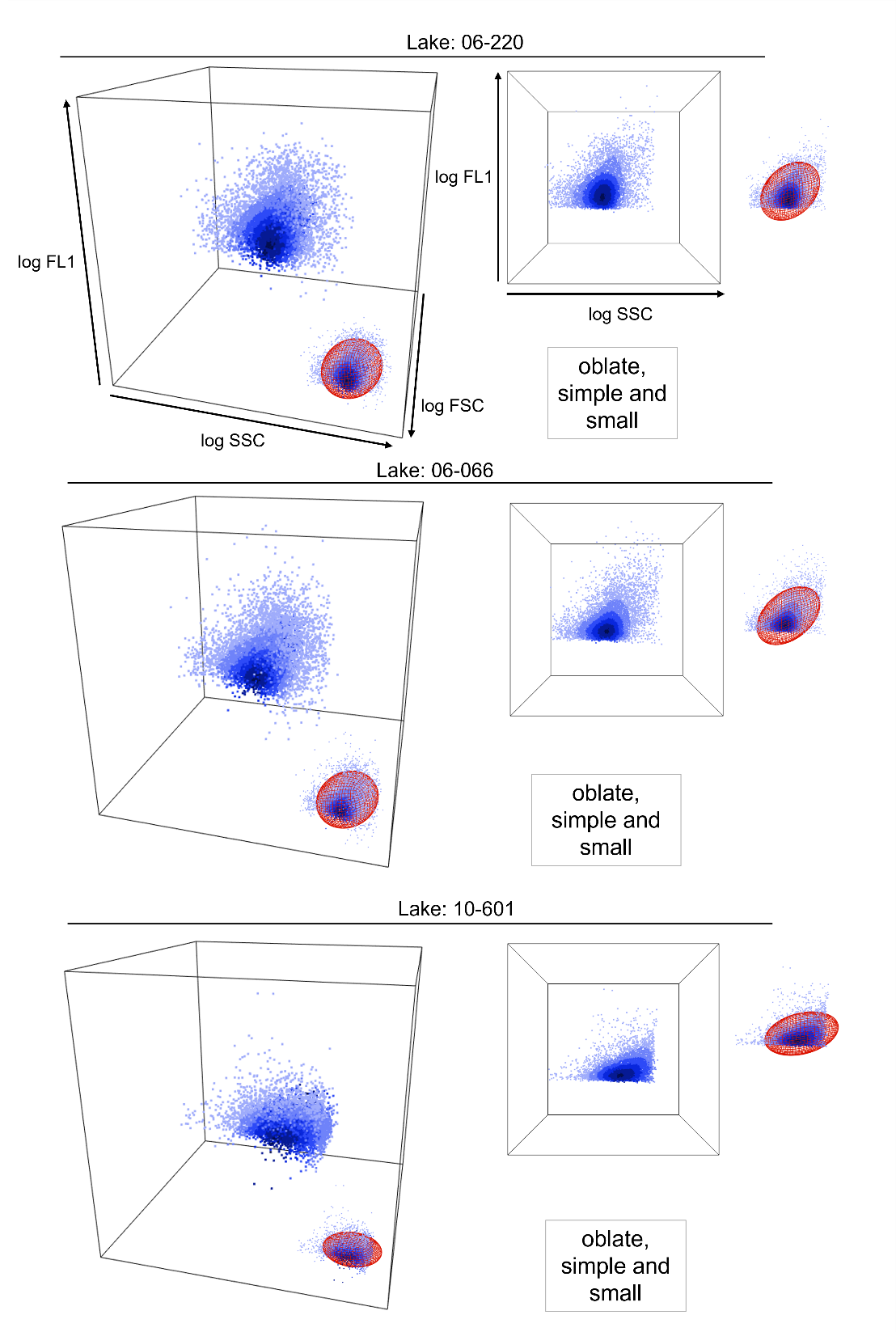


**Figure S2**: Examples of dot plots with their corresponding ellipsoids illustrating oblate shapes.


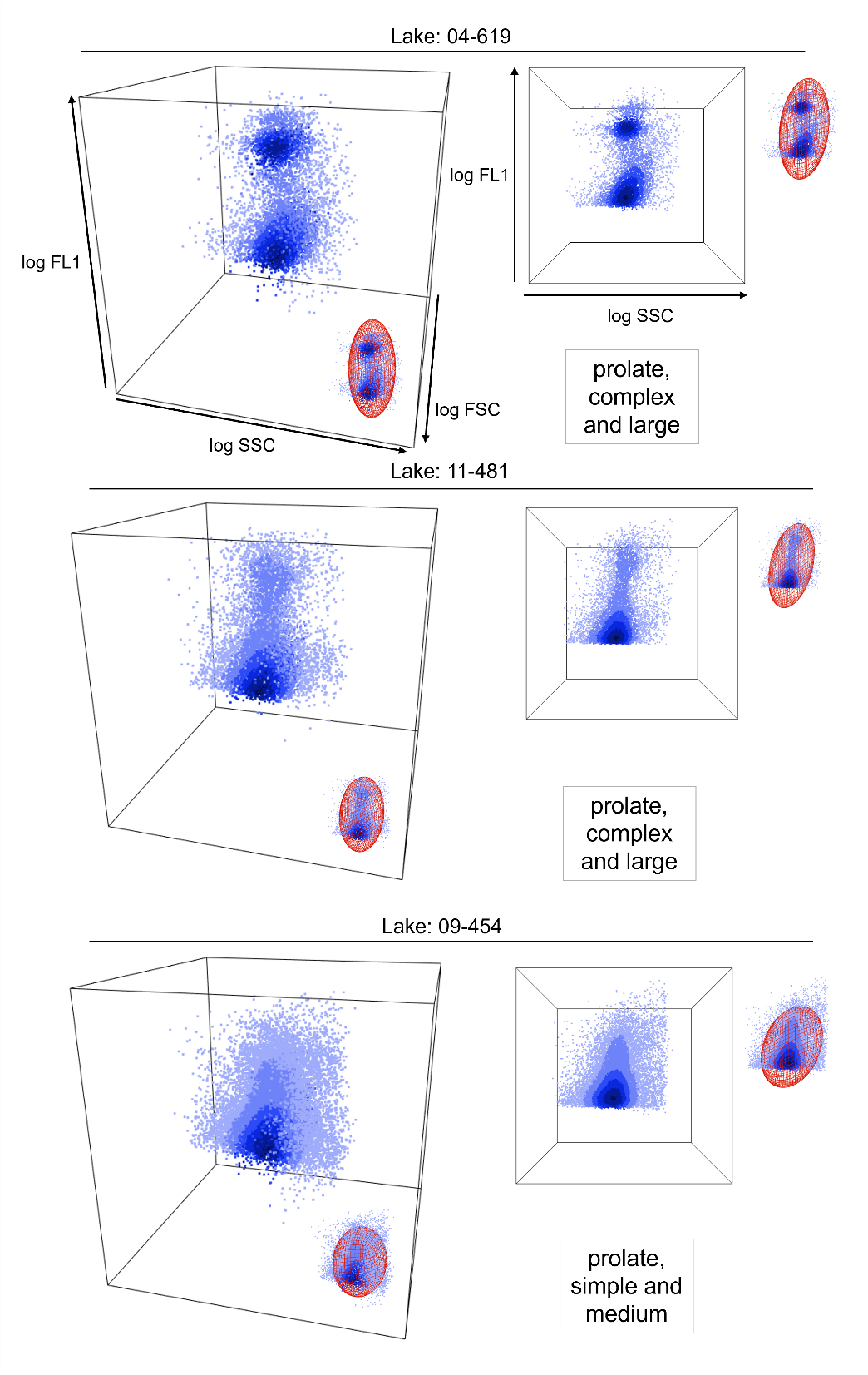


**Figure S3**: Examples of dot plots with their corresponding ellipsoids illustrating prolate shapes


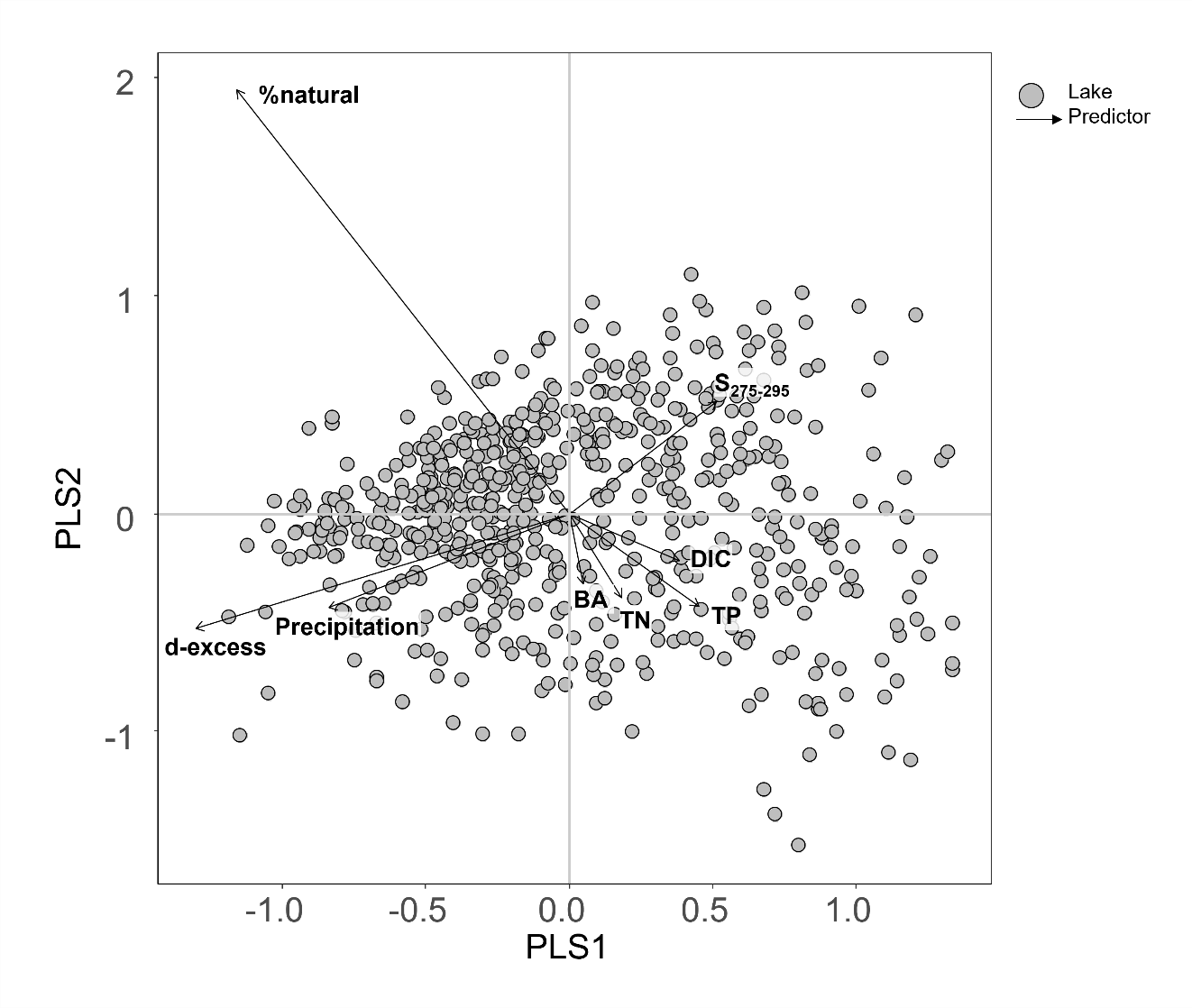


**Figure S4:** The first two components extracted from the Partial least-squares (PLS) regression analysis based on the selected environmental variables and the selected cytometric metrics. The length of the arrows represents the importance of the environmental predictors, i.e. watershed fraction cover by natural landscape (%natural), d-excess, precipitation, bacterial abundance (BA), total nitrogen (TN) concentration, total phosphorus (TP) concentration, dissolved inorganic carbon (DIC) concentration and spectral slope of the coloured dissolved organic matter for the wavelength interval of 275 to 295 nm (S_275-295_).


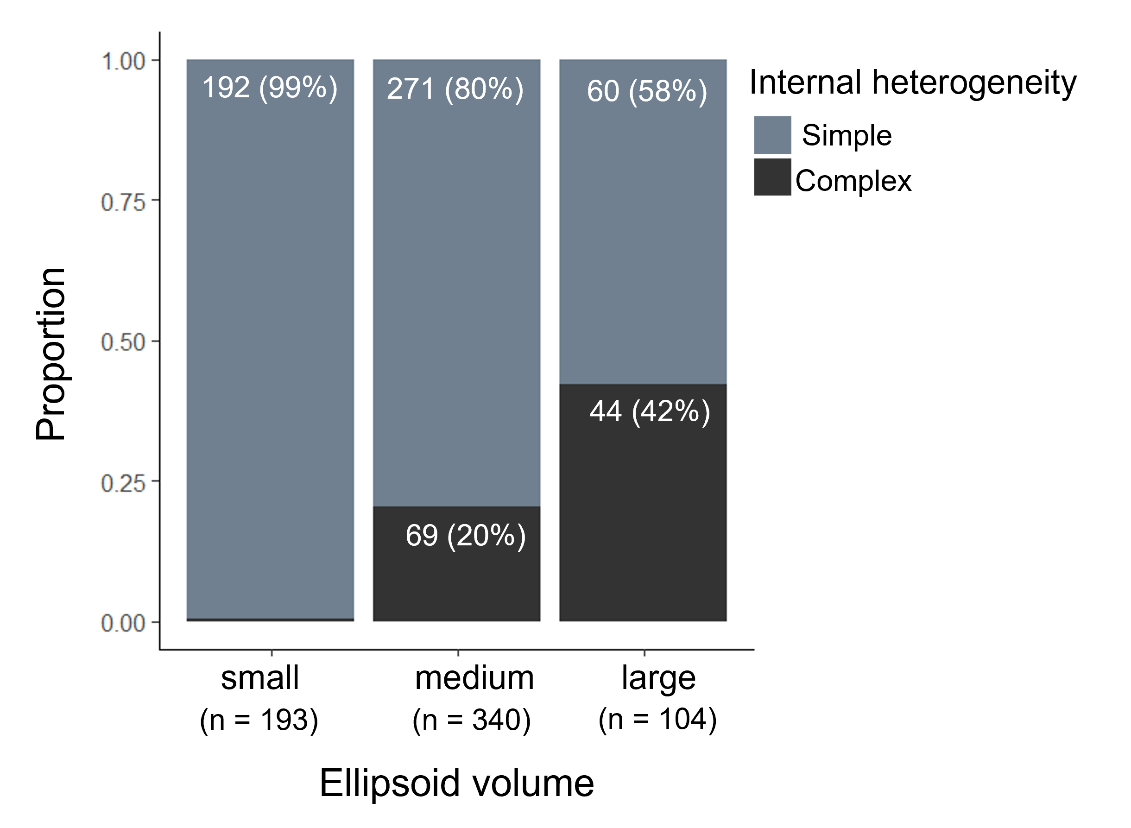


**Figure S5: Ellipsoid volume vs internal heterogeneity.** Proportion of the number of lakes based on ellipsoid volume (small, medium and large) and internal heterogeneity (simple and complex) of the BMS.


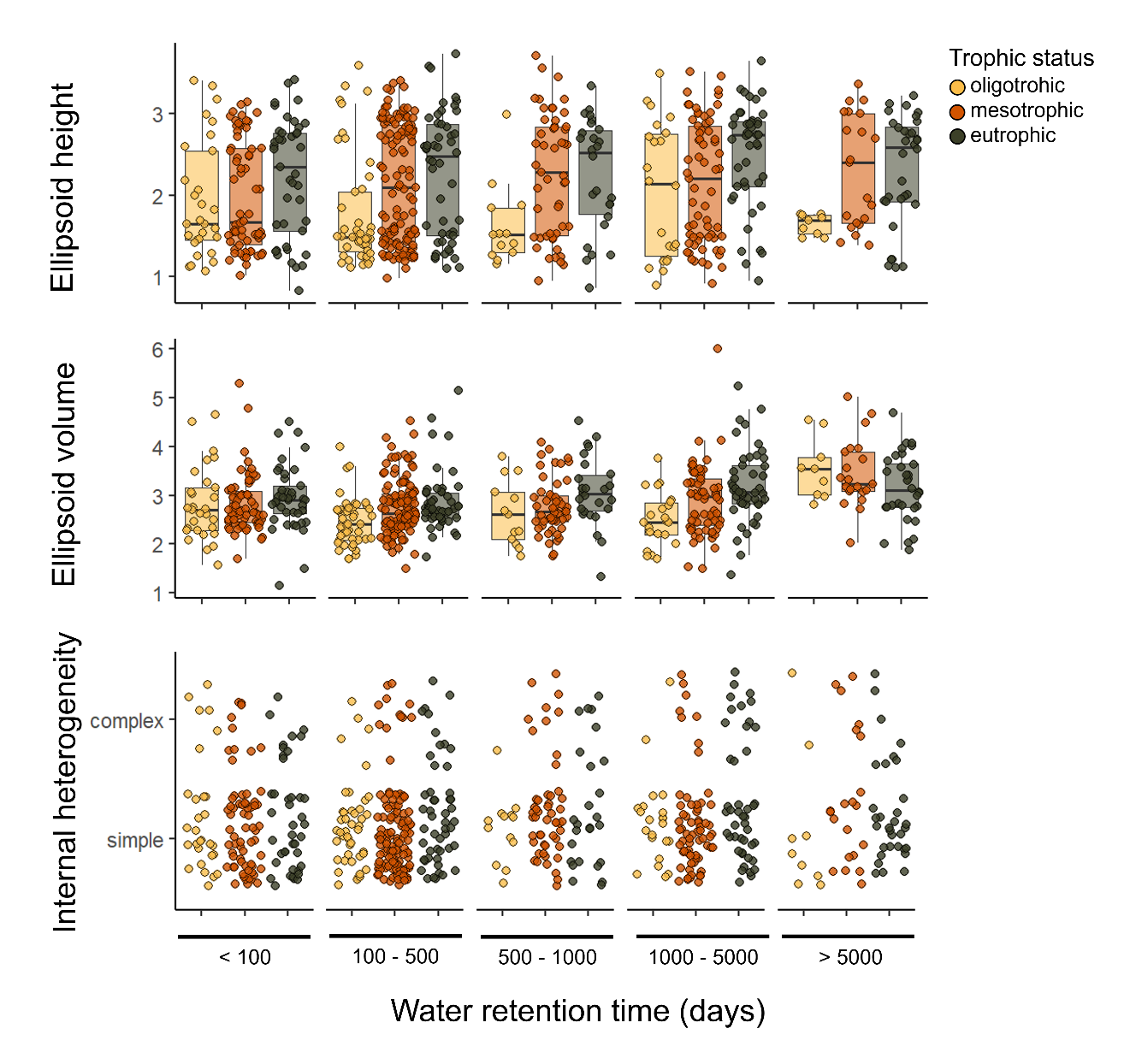


**Figure S6: Patterns of ellipsoid height, ellipsoid volume and cytometric populations of the bacterioplankton morphological structure along water retention time and trophic status gradients**. Lakes are grouped by categories of water retention time (i.e. < 100, 100 – 500, 500 – 1000, 1000 – 5000 and > 5000), with trophic status (i.e. oligotrophic, mesotrophic, eutrophic) nested within each category. Boxplots display the 10th, 25th, 50th (median), 75th and 90th percentiles. Data points are coloured by trophic status


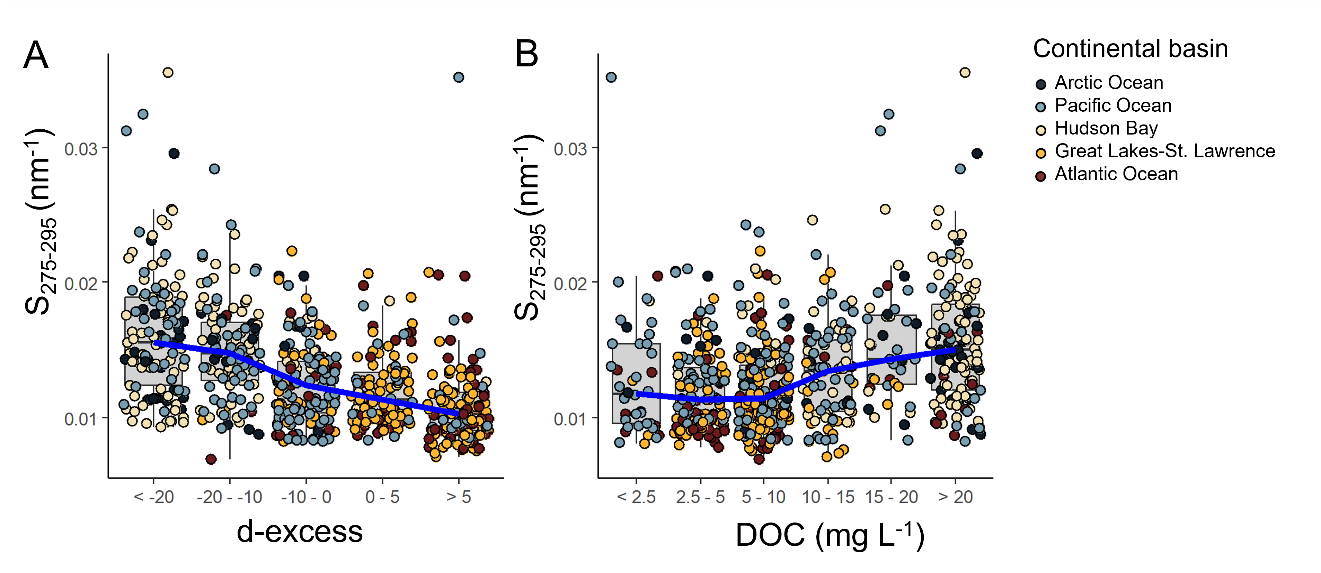


**Figure S7: Patterns of environmental variables.** Box plots showing the distribution of the lakes based on categories of **A)** d-excess vs S_275-295_ (nm_-1_) values and **B)** dissolved organic carbon (DOC) concentration vs S_275-295_ (nm_-1_) values. Lower to upper values are indicated, respectively, 10%, 25%, 50% (median), 75% and 90%. Data points are coloured by the continental basins.


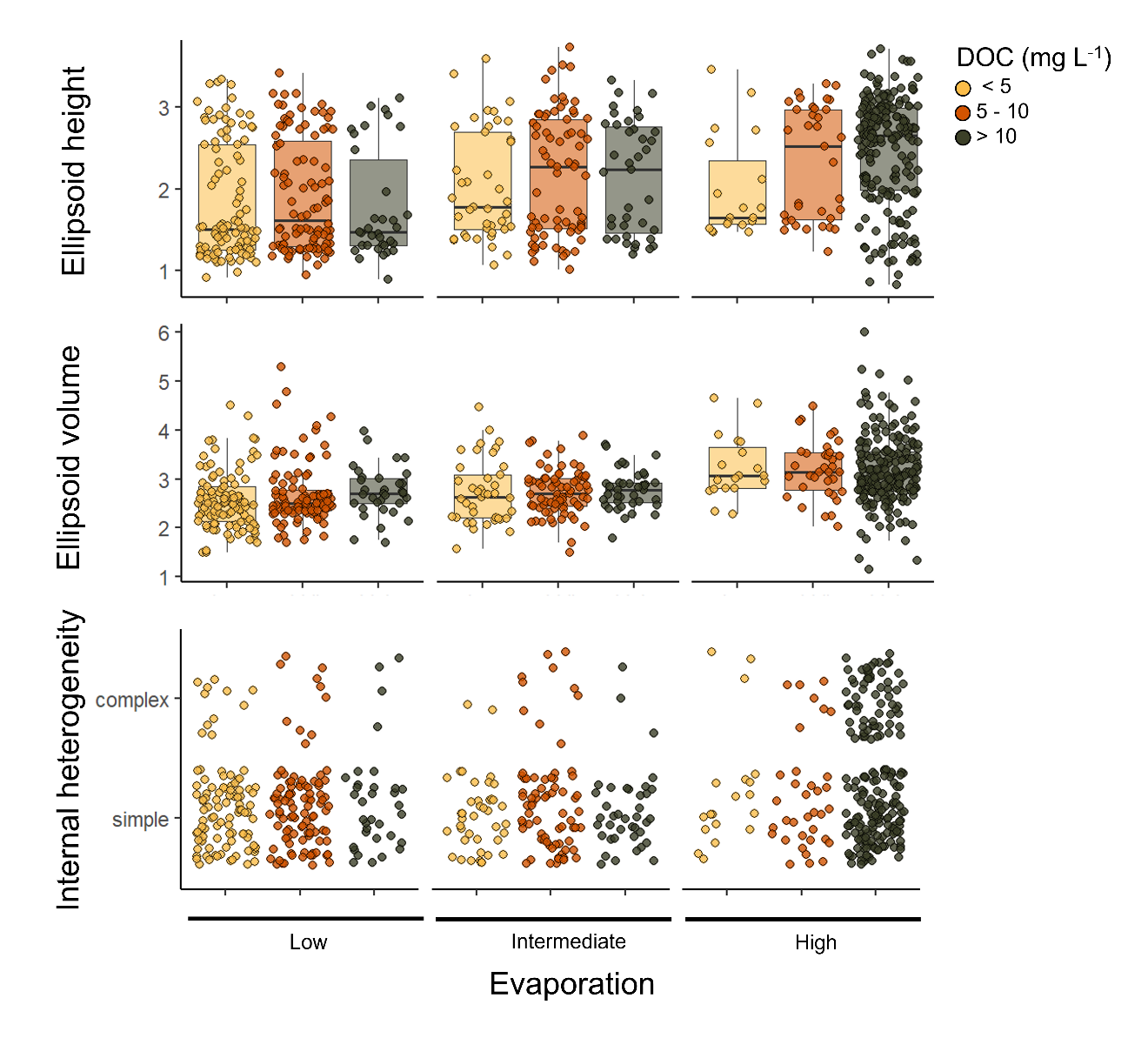


**Figure S8: Patterns of ellipsoid height, ellipsoid volume and cytometric populations of the bacterioplankton morphological structure along hydrologic and dissolved organic carbon (DOC) concentration gradients**. Lakes are grouped by degree of evaporation (low, intermediate, high), with DOC concentration (i.e. < 5, 5 - 10 and > 10 mg L^-1^) nested within each category. Boxplots display the 10th, 25th, 50th (median), 75th and 90th percentiles. Data points are coloured by DOC concentration.


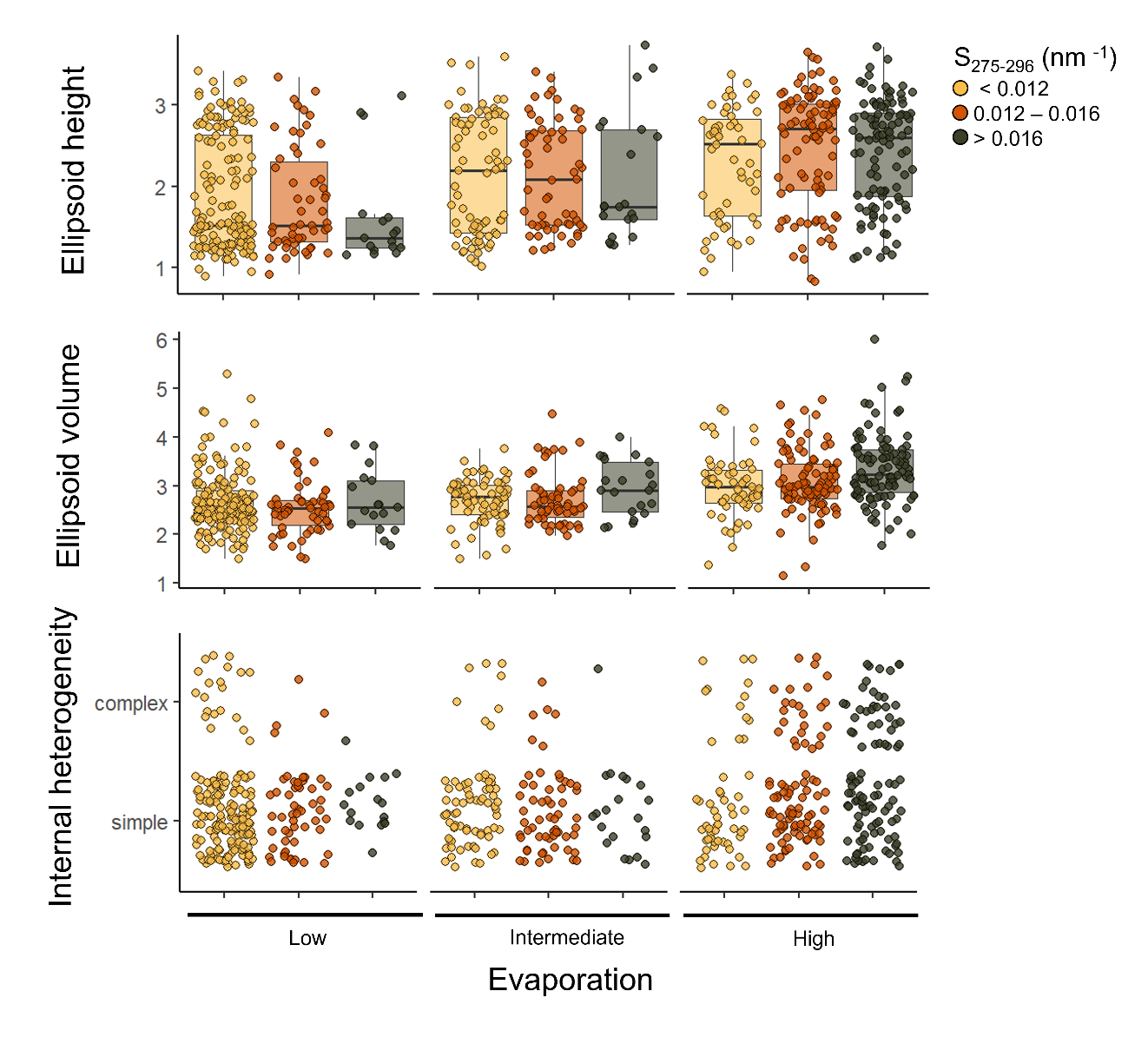


**Figure S9: Patterns of ellipsoid height, ellipsoid volume and cytometric populations of the bacterioplankton morphological structure along hydrologic and dissolved organic carbon (DOC) concentration gradients**. Lakes are grouped by degree of evaporation (low, intermediate, high), with spectral slope of coloured dissolved organic matter between 275 and 295 nm (S_275-295_) (i.e. < 0.012, 0.012 – 0.016 and > 0.016 nm^-1^) nested within each category. Boxplots display the 10th, 25th, 50th (median), 75th and 90th percentiles. Data points are coloured by S_275-295_ categories.
